# The impact of nanoparticle aggregation on their size exclusion during transport in porous media: One- and three-dimensional modelling investigations

**DOI:** 10.1038/s41598-019-50493-6

**Published:** 2019-10-01

**Authors:** Peyman Babakhani

**Affiliations:** School of Engineering, University of Liverpool, Liverpool, Merseyside L69 3GH UK

**Keywords:** Environmental impact, Hydrology

## Abstract

Greater particle mobility in subsurface environments due to larger size, known as size exclusion, has been responsible for colloid-facilitated transport of groundwater contaminants. Although size exclusion is not expected for primary engineered nanoparticles (NP), they can grow in size due to aggregation, thereby undergoing size exclusion. To investigate this hypothesis, an accurate population balance modelling approach and other colloid transport theories, have been incorporated into a three-dimensional transport model, MT3D-USGS. Results show that incorporating aggregation into the transport model improves the predictivity of current theoretical and empirical approaches to NP deposition in porous media. Considering an artificial size-variable acceleration factor in the model, NP breakthrough curves display an earlier arrival when aggregation is included than without. Disregarding the acceleration factor, aggregation enhances NP mobility at regions close to the injection point at a field scale and causes their retention at greater distances through alteration of their diffusivities, secondary interaction-energy minima, and settling behaviour. This results in a change of residual concentration profiles from exponential for non-aggregating dispersions to non-monotonic for aggregating dispersions. Overall, aggregation, hitherto believed to hinder the migration of NP in subsurface porous media, may under certain physicochemical conditions enhance their mobilities and deliver them to further distances.

## Introduction

The growing use of nanoparticles (NP) in consumer products along with other attractive engineered applications such as environmental remediation^1–5^, enhanced petroleum reservoir recovery^6–8^, and agricultural fertilizers/pesticides^9–11^, can eventually lead to NP release into the environment^12,13^. Due to concerns about uncontrolled spread of NP, the use of nanotechnology for environmental applications such as groundwater remediation has already been restricted by regulatory in certain countries such as the UK, whereas in many other countries, such as the USA, environmental application of nanotechnology at a field scale is already a common practice^14,15^.

The role of natural colloidal particles in the transport of solute contaminants has been demonstrated in many studies^16–19^. Being a colloid and having considerable capability for uptake of dissolved contaminants, NP aggregates once released into subsurface environments can crucially act as carriers of hazardous contaminants such as radionuclide, particularly if NP dispersions are purposefully injected into groundwater for the remediation of such pollutants^1,20,21^. The most notable phenomenon responsible for the mobility of colloidal particles and their associated contaminants has been ‘size exclusion’^22–24^, which is the exclusion of particles from pores and stagnant domains within the media that are smaller than the size of particles or are less accessible to them^23,24^. This allows larger particles to remain within main flow streams which have a higher velocity than the average groundwater velocity, giving rise to particle mobilities higher than expected^25,26^. Such size exclusion may manifest in the experimental breakthrough curve (BTC) of porous media as an acceleration or early arrival of the curve.

Nanoparticles are generally not expected to undergo size exclusion due to their small size leading to higher diffusion, allowing them to diffuse more easily into low mobility zones of subsurface media. This may cause NP trapsort to show a retardation rather than an acceleration behvior^22,27,28^. However, under environmental conditions NP can grow in size due to aggregation, especially within porous media^22,29^. This can reduce then NP mobility in porous media through different deposition mechanisms, namely attachment, straining, and ripening^30–33^. Several experimental observations of BTC early arrival from packed columns have indicated that size exclusion may also occur for NP aggregates^34–38^. Nevertheless, there is no clear understanding of how size exclusion emerges in BTCs when other concurrent transport mechanisms also occur. Although field-scale implementations of NP to date have shown migration distances of less than few meters, recently the injection of zero valent iron NP into a fractured chalk aquitard with moderate salinity revealed that, in certain physicochemical condition, NP can migrate a distance of 47 m^39^. In that study, earlier arrival of NP aggregates than a conservative solute was observed at low stabilizer concentration which was attributed to size exclusion. However, the role of concurrent aggregation with transport in such behaviour was not investigated or clarified^39^. To date there has not been any systematic investigation on the impact of concurrent aggregation on accelerating mechanisms of NP transport such as size exclusion. If aggregation triggers size exclusion, it follows that in certain physicochemical conditions within the subsurface environment, NP aggregates can uncontrollably migrate to drinking water resources in the vicinity of NP injection sites or NP aggregates detached from hotspots in the waste stream can reach distances further than expected, posing toxicity risks of both NP aggregates and contaminants adsorbed on their surfaces to human health^2,40^.

**Figure 1:**
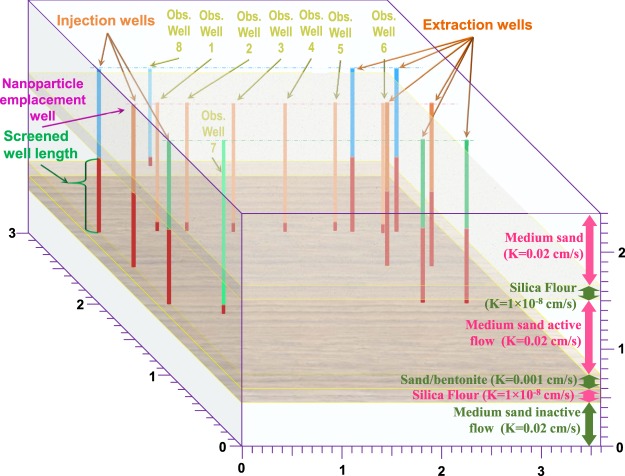
Field-scale model domain used in 3-D simulations following Johnson *et al*.^31^.

Accurate modelling tools can aid regulatory assessment of potential risks for NP release into the environment and help practitioners design NP application strategies at environmentally-relevant scales^22,41^. For such purposes, continuum models, commonly known as advection-dispersion-equation (ADE) solved over continuous spatial and temporal domains of the bulk system, may be promising due to their ability in describing several concurrent transport mechanisms in porous media and their scalability^22^. Although there have been a few studies^42–46^ to date considering aggregation in a porous media transport models, they were limited to 1-D modelling^42–44,46^, except one^45^ which upscaled the model to 2-D but did not consider particle size distribution dynamics in the model. Besides, consideration of aggregation in those studies have been focused on simplifying the approach to aggregation rather than incorporating an accurate and computationally affordable population balance model. The present study’s model development is the first to incorporate an accurate number-concentration-based population balance equation representing NP aggregation, into a 3-D field-scale continuum model for NP transport^47^. Furthermore, the present study is the first systematic investigation of the impact of NP aggregation on their size exclusion during transport in porous media. The model domains scrutinized include a1-D, laboratory-scale domain and a 3-D, multi-layered, field-scale domain taken from Johnson *et al*.^31^ as shown in Fig. 1. After model validation against previous aggregation codes^48^ and against experimental data from literature^49^, the manifestation of aggregation and size exclusion phenomena in the BTC shape and the residual concentration profile (RCP) is thoroughly investigated. Hydroxyapatite (HAp) NP are used as a model NP due to their promising applications in remediation of groundwater contaminated with radionuclide^4,20,50,51^, and their potential for being used as agricultural fertilizer^11^.

## Mathematical Model

For combination with the continuum model, a modelling approach to aggregation known as the fixed pivot (FP) technique^52^ was chosen due to its flexibility in selecting the particle volume/size discretization. Fixed pivot is a well-established accurate population balance modelling approach to aggregation-driven particle size distribution (PSD) evolutions^48,52,53^, already used for modelling experimental data of HAp NP aggregation and sedimentation in aqueous solutions both under quiescent condition^48^ and dynamic (rotating) condition^29^. The FP model equations are as follows^52^:

1 $$\frac{d{n}_{k}}{dt}=\mathop{\sum }\limits_{\begin{array}{c}j,i\\ {v}_{k-1}\le ({v}_{j}+{v}_{i})\le {v}_{k+1}\end{array}}^{j\ge i}[1-\frac{1}{2}{\delta }_{j,i}]{\eta }_{k}{\alpha }_{ag{g}_{j,i}}{\beta }_{j,i}\,{n}_{j}{n}_{i}\,-{n}_{k}\sum _{i}{\alpha }_{ag{g}_{k,i}}\,{\beta }_{k,i}\,{n}_{i}$$

where *n*_*k*_ is the particle number concentration of agglomerates in size class *k* [*L*^−3^], *β* is the collision frequency given in the Supplementary Information (SI), *α*_*agg*_ is the attachment efficiency for particle-particle collisions, *v*_*i*_ is the volume of solids within each aggregate in size class *i*, *δ* is Kronecker’s delta, i,j,k are size class subscripts, and *η*_*k*_ is:

2 $$\begin{array}{ccc}{\eta }_{k} & = & \{\begin{array}{c}\frac{{v}_{k+1}-({v}_{j}+{v}_{i})}{{v}_{k+1}-{v}_{k}},{v}_{k}\le ({v}_{j}+{v}_{i})\le {v}_{k+1}\\ \frac{({v}_{j}+{v}_{i})-{v}_{k-1}}{{v}_{k}-{v}_{k-1}},{v}_{k-1}\le ({v}_{j}+{v}_{i})\le {v}_{k}\end{array}\end{array}$$

The Brinkman permeability model for collision frequencies, and a power-law expression for settling velocity inducing differential sedimentation collisions were also selected based on their promising applications in describing aggregation and settling behaviours of HAp NP in the previous study^48^.

The governing equation for continuum model of nanoparticle transport in porous medium for size class *k* in a 3-D transient flow may be written as follows^22,54^:

3 $$\varepsilon \frac{\partial {C}_{k}\,}{\partial t}({\vartheta }_{k})=\frac{\,\partial }{\partial {x}_{L}}(\varepsilon {D}_{L,T}\frac{\partial {C}_{k}\,}{\partial {x}_{T}})-\frac{\partial (\varepsilon {V}_{L}{C}_{k})\,}{\partial {x}_{L}}-\varepsilon {K}_{at{t}_{k}}{C}_{k}$$

where *x*_*L*_ and *x*_*T*_ are the distances [L] along the respective Cartesian coordinate axis, *L* and *T*, in porous media, *t* is time elapsed [T], *C*_*k*_ is the mass concentration [ML^−3^] of fluid-phase particles for size class *k*, *V*_*L*_ is the pore water velocity along the coordinate axis *L* [L T^−1^], *ε* is the porosity of porous media [−], *ρ*_*b*_ is the porous medium bulk density [ML^−3^], *D*_*L,T*_ is the dispersion coefficient tensor [L^2^T^−1^] described elsewhere^54^, ${K}_{att{}_{k}}$ is the attachment rate coefficient [T^−1^] for size class *k* as determined in the SI, and *ϑ*_*k*_ is the acceleration factor (≤1) for size class *k* given as^22,55,56^:

4 $${\vartheta }_{k}=1+\frac{{\rho }_{b}}{\varepsilon }{K}_{{d}_{k}}$$

where *K*_*dk*_ is a coefficient (≤0) herein used to control the acceleration factor for each size class *k*. Consideration of detachment is described in the SI.

## Results and Discussion

### Model validation

The modified MT3D-USGS was validated for pure aggregation by comparing its results with those of the MATLAB code developed and validated in a previous study^48^ for NP aggregation and sedimentation in quiescent aqueous solutions. Here the comparison of the two codes was carried out by turning off all reaction terms in the governing equation of the modified MT3D-USGS code, allowing only the transport (advection/dispersion) and the aggregation mechanisms to be operative within a 1-D domain. For a synchronized duration, MATLAB code calculating aggregation driven size evolution under quiescent (static) condition, should at the beginning of the BTC plateau match the modified MT3D-USGS code calculating aggregation driven size evolution in porous media.

The result of this comparison, shown in Supplementary Fig. S1, demonstrates that PSDs computed by the two codes agree well for various ranges of attachment efficiencies and fractal dimensions. Slight discrepancies between the two codes observed at peaks of PSDs may relate to interference from the dispersion mechanism in the transport model results. Similar comparisons are performed for evolutions of mean hydrodynamic diameter, *D*_*H*_, over time calculated by the modified MT3D-USGS at the outlet of the packed column and calculated by the previously developed MATLAB code for the static aqueous media. As shown in Supplementary Fig. S2 the resulted curves meet at the beginning of the BTC plateau in all cases. After this stage, *D*_*H*_ curves for the static condition keep growing while those of the transport model remain constant due to the same aggregation time (residence time) for all aggregates transported through the porous media system. The mass balance errors calculated by the modified MT3D-USGS was less than 0.1% in all simulations. These results demonstrate the validity of the modified MT3D-USGS code.

### Prediction performance of the model

**Figure 2:**
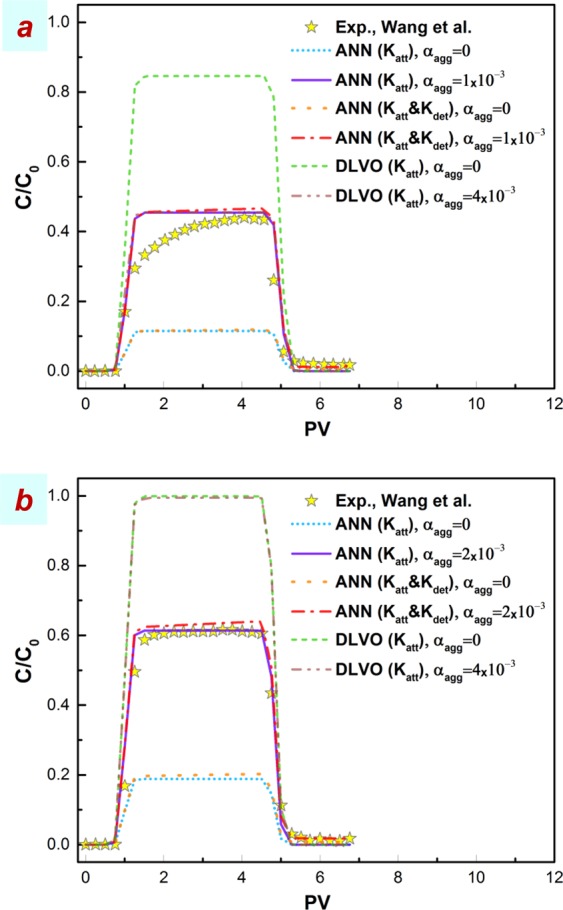
Comparison of the modified MT3D-USGS model outcomes with experimental data of Wang *et al*.^49^ at two chemical conditions: (**a**) 50 mM KCl (**b**) 0.5 mM CaCl_2_. The full experimental details are summarized in Supplementary Table S1. The modelling approaches include the use of CFT-DLVO and ANN based correlations for predicting attachment (*K*_*att*_) and detachment (*K*_*det*_) rate coefficients.

Column experiment data of Wang *et al*.^49^ at two electrolyte concentrations of 50 mM KCl and 0.5 mM CaCl_2_ were used to assess the predictivity of the model when combined with two approaches of colloid filtration theory (CFT)^57,58^ coupled with Derjaguin, Landau, Verwey, and Overbeek (DLVO)^59–61^ designated herein as CFT-DLVO and artificial neural network (ANN)-based correlations^62^. As shown in Fig. 2, incorporating aggregation into the model by increasing *α*_*agg*_ from zero to 1 × 10^−3^ and 4 × 10^−3^ results in better predictions by both approaches. Babakhani *et al*.^62^ using ANN overpredicted the same experimental BTC plateau height at 50 mM KCl (*R*^2^ = 0.432) whilst underpredicting that at 0.5 mM CaCl_2_ (*R*^2^ = 0.738) using a constant attachment coefficient with size and without considering aggregation. Considering a variable attachment efficiency with size classes, the use of the ANN-based correlations in the present study, underpredicts the BTC plateau in both cases more noticeably (*R*^2^ *<* 0) than the previous study (Fig. 2a,b). However, when aggregation is included in the model, the experimental BTC plateau heights are reproduced well at both 50 mM KCl (*R*^2^ = 0.900) and 0.5 mM CaCl_2_ (*R*^2^ = 0.985). It should be noted that the slope of BTC plateau at 50 mM KCl is not expected to be reproduced by the current model taking aggregation and deposition mechanisms into account since this slope may be related to the blocking phenomenon which is beyond the scope of the present study^22,63^.

In opposition to ANN correlations, CFT-DLVO without considering aggregation in the model overpredicts the height of BTC plateau (*R*^*2*^ < 0). This discrepancy may not be because of ignoring the electrosteric repulsion interaction energy emanated from the presence of humic acid (10 mg/L) within experimental HAp dispersions, since considering such a repulsive force in the model should reduce NP attachment thereby elevating the BTC height^64^. When aggregation is incorporated, the model predicts the height of the BTC well at 50 mM KCl (*R*^*2*^ = 0.889), whereas at 0.5 mM CaCl_2_ it does not show a discernible impact on the modelled BTC due probably to the role of bridging interactions in the presence of divalent cation (CaCl_2_) and polymer (humic acid) in the present extended DLVO calculation, not being included^62,65^. Overall, these results suggest that incorporating the aggregation mechanism into NP transport continuum models can substantially improve the predictivity of current approaches, such as the DLVO theory and ANN empirical correlations.

### The impact of aggregation on size exclusion in 1-D transport

**Figure 3:**
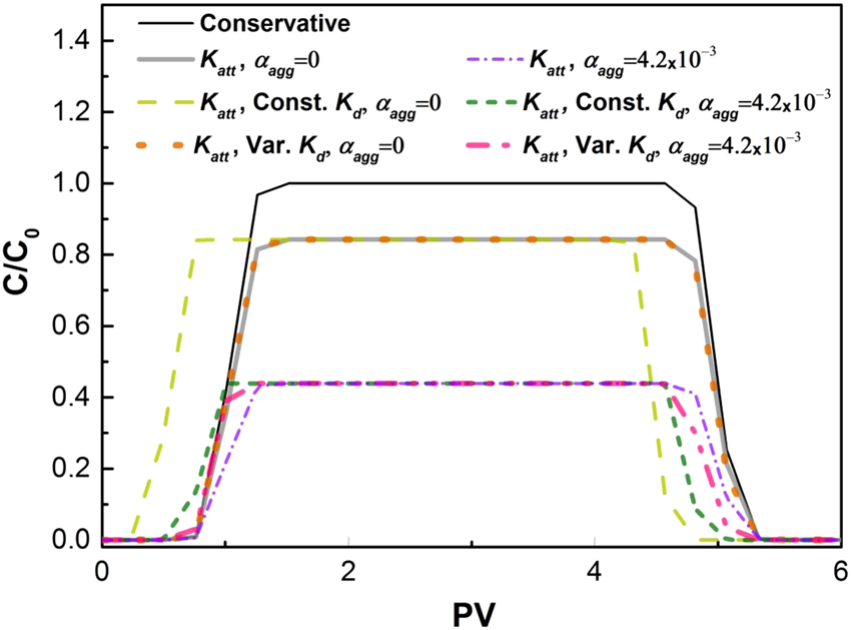
Model-produced BTC for NP transport at 1-D scale based on different scenarios: aggregation included (*α*_*agg*_ ≠ 0) or not included (*α*_*agg*_ = 0) and acceleration not included, assumed constant (Const. *K*_*d*_), or assumed variable (Var. *K*_*d*_). The attachment rate coefficient (*K*_*att*_) is determined from the CFT-DLVO. Other model characteristics are selected based on the experimental data of Wang *et al*.^49^ at an electrolyte concentration of 50 mM KCl as listed in Supplementary Table S1.

Using the validated model, different scenarios for involving aggregation and size exclusion were investigated based on characteristics of the 1-D model at an electrolyte concentration of 50 mM KCl. Hereafter, only CFT-DLVO is used to estimate the attachment rate coefficient. Results of this analysis, shown in Fig. 3, demonstrate that attachment generally reduces the BTC plateau height (*C/C*_0_) from around 1 to around 0.8 (Fig. 3). When a constant acceleration factor is also included in the model through a negative *K*_*d*_, without considering aggregation (*α*_*agg*_ = 0) both rising and falling limbs of the BTC emerge earlier than those of BTCs with only advection, dispersion and/or attachment mechanisms. When aggregation is considered by increasing *α*_*agg*_ from zero to 4.2 × 10^–3^, the height of the BTC reduces substantially (*C/C*_0_ ≈0.4). This is due to particle size growth enhancing their deposition since size growth increases the depth of the secondary interaction energy well, shown by sphere-plate interaction energy profiles in Supplementary Fig. S3, thereby promoting particle attachment; consistent with previous studies mentioning that aggregation in porous media can enhance their deposition^30,46^. This may also be due to an increase in aggregate settling velocity related to an increase in their size and density, with a subsequent increase in their retention within porous media. This links with a further mechanism in the transport of colloidal particles from the pore fluid to the vicinity of porous media grains, known as interception which occurs when a particle comes into contact with the porous media grains due to its finite size^48,58,66,67^. These are all considered in the present modelling through CFT-DLVO theories. Despite a decrease in the height of the BTC, it is evident that both rising and falling limbs of BTC arrive earlier than those of conservative BTC (Fig. 3), consistent with the experimental investigations of concurrent aggregation and transport of TiO_2_ NP in packed columns by Solovitch *et al*.^37^ concluding that NP aggregation within porous media enhances their deposition, while aggregates which survive retention can undergo size exclusion. In another scenario, a size-variable *K*_*d*_ was considered (Supplementary Table S1) so that it yields the same BTC as that under only deposition mechanism. In this scenario, the inclusion of aggregation led to a considerable early arrival of BTC is observed especially for the falling limb of BTC (Fig. 3). These results suggest that under physicochemical conditions where NP is prone to size exclusion, their aggregation within porous media can accelerate their migration despite giving rise to their attachment and removal as well.

### The impact of aggregation on size exclusion in 3-D transport

**Figure 4:**
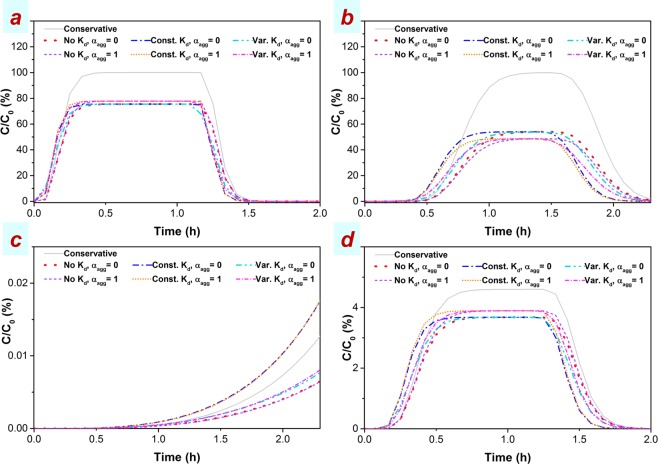
Breakthrough curves obtained at wells #2 (**a**), #4 (**b**), #7 (**c**), and #8 (**d**) at 4.5 g/L injection concentration of HAp NP into the 3-D model domain. Different scenarios comprise of aggregation included (*α*_*agg*_ ≠ 0) or not included (*α*_*agg*_ = 0) and acceleration not included (No *K*_*d*_), assumed constant (Const. *K*_*d*_), or assumed variable (Var. *K*_*d*_). The attachment rate coefficient (*K*_*att*_), used in all scenarios other than the conservative transport, which is determined from the CFT-DLVO. Other model characteristics are listed in Supplementary Table S2.

The transport of HAp NP within the 3-D model domain generally shows a high mobility (Fig. 4 and Supplementary Fig. S4). Breakthrough curves resulted from different scenarios at wells #2, #4, #7, and #8 are compared in Fig. 4 demonstrating that for wells located close to the injection point (<0.87 m), i.e, wells #2, #7, #8, incorporation of aggregation leads to an increase in the height of the BTC plateau (Fig. 4a,c,d) contrary to 1-D simulations showing a decrease in all BTC heights with incorporation of aggregation mechanism. In such cases although including the acceleration factor generally leads to early BTCs, the addition of aggregation does not give rise to early arrival of BTCs where a size-variable acceleration factor is considered, with such cases exhibiting a slight retardation in BTC. These results suggest that manifestations of transport phenomena in BTCs of NP being transported within complex 3-D heterogeneous domains, especially at marginal regions of the mainstream flow and within low-permeable layers, may not necessarily follow those of simplified 1-D domains. This is in agreement with the study of Phenrat *et al*.^68^ demonstrating that within a multi-layered 2-D porous media, transport of zerovalent iron NP followed preferential flow paths. However, at wells further from the injection point (1.5 m) and located on streamlines of the main flow direction (wells #4), aggregation leads a reduction in the BTC height (Fig. 4b) similar to 1-D simulations. In this case including a size-variable acceleration factor yields a considerable earlier arrival when the aggregation mechanism is added, when compared to the case with no aggregation—in line with 1-D modelling results. This early arrival is more significant for the falling limb than the rising limb of the BTC since size growth due to aggregation, which is a time-dependent mechanism, is higher for the falling limb than that of the rising limb.

Observation wells at the margin of the main active-flow area, e.g., well #7 (Fig. 4c) at the bottom sand-bentonite layer and well #8 (Fig. 4d) just below the upper impermeable silica flour layer, show generally lower *C/C*_0_, less than 0.02% and < 5%, respectively, in all scenarios compared to wells at the central part of the active-flow region. However, these concentrations (on the order of 1 mg/L) are still environmentally and eco-toxicology considerable^69^. This is consistent with previous studies^45,68^ showing that despite relatively low flow velocity in low-permeability layers, NP still enter those layers and are deposited in stagnation zones.

**Figure 5:**
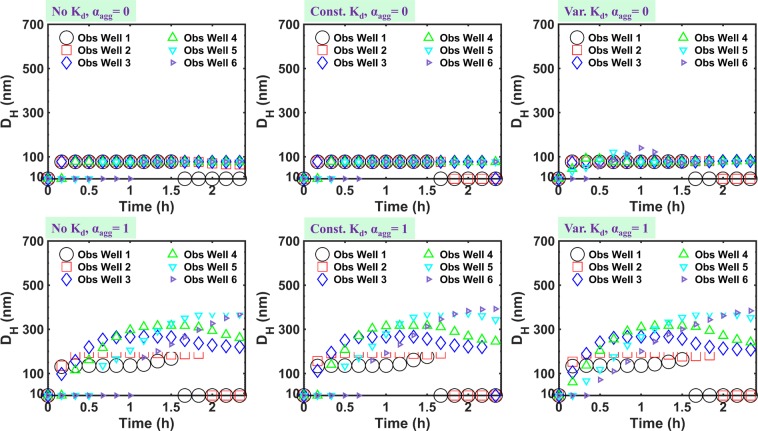
Model-produced *D*_*H*_ at different observation wells of the 3–D model domain for various scenarios of aggregation included (*α*_*agg*_ ≠ 0) or not included (*α*_*agg*_ = 0) and acceleration not included (No *K*_*d*_), assumed constant (Const. *K*_*d*_), or assumed variable (Var. *K*_*d*_).

Investigating mean hydrodynamic diameter growth over time as shown in Fig. 5, reveals that when aggregation is included, the change in size is not considerable at wells #1 and #2 while it becomes significant at wells #3 to #6, where size trends exhibit a peak corresponding to the centre of the BTC but, with a more gradual rise and fall compared to the BTC. Without aggregation, when a size-variable acceleration factor is considered, a slight increase in the mean size is observed at wells further from the injection point (wells #5, #6) after ~1 h. This is interpreted as the early arrival of particles in larger size classes of the initial PSD to these wells, thereby increasing the local concentration of these size classes leading to a slight increase in the geometrically mass-averaged diameter.

Breakthrough curves of aggregates versus PSD evolution are shown in Supplementary Figs, S5-S8 for wells #1, #4, #7, and #8, respectively. These graphs almost consistently display earlier arrival of NP aggregates in intermediate size classes compared to those in larger size classes whether acceleration is included or not. This may be due to attachment of larger aggregates to porous media grains upon growth of particles from middle-size classes to larger ones during aggregation due to increase in their secondary energy well depth as described already. This removal of particles from larger size classes upon their growth may lead to maintaining a so-called ‘local dynamic equilibrium’ for relatively larger size classes^45,70–72^. To investigate this further the RCPs were investigated.

### Retained concentration profiles in 3-D transport

While an exponentially decaying RCP has been attributed to colloid deposition mechanisms described by the CFT model^73^ and a hyper-exponential shape of RCP has been attributed to the straining mechanism^74^, the reason for non-monotonic RCPs remains controversial^22^. A variety of factors, e.g., heterogeneities^75^, presence of polymers^76^, release/re-entrapment of aggregates^77^, etc., have been proposed to be responsible for such a behaviour of RCP, and various models have been tried to describe this trend. Although without providing a strong evidence it has been suggested that aggregation in the suspended phase of particles in porous media may be the key mechanism in shaping non-monotonic RCPs^22,78,79^. Such a hypothesis can only be proved using an accurate model which considers both concurrent aggregation and transport of NP.

**Figure 6:**
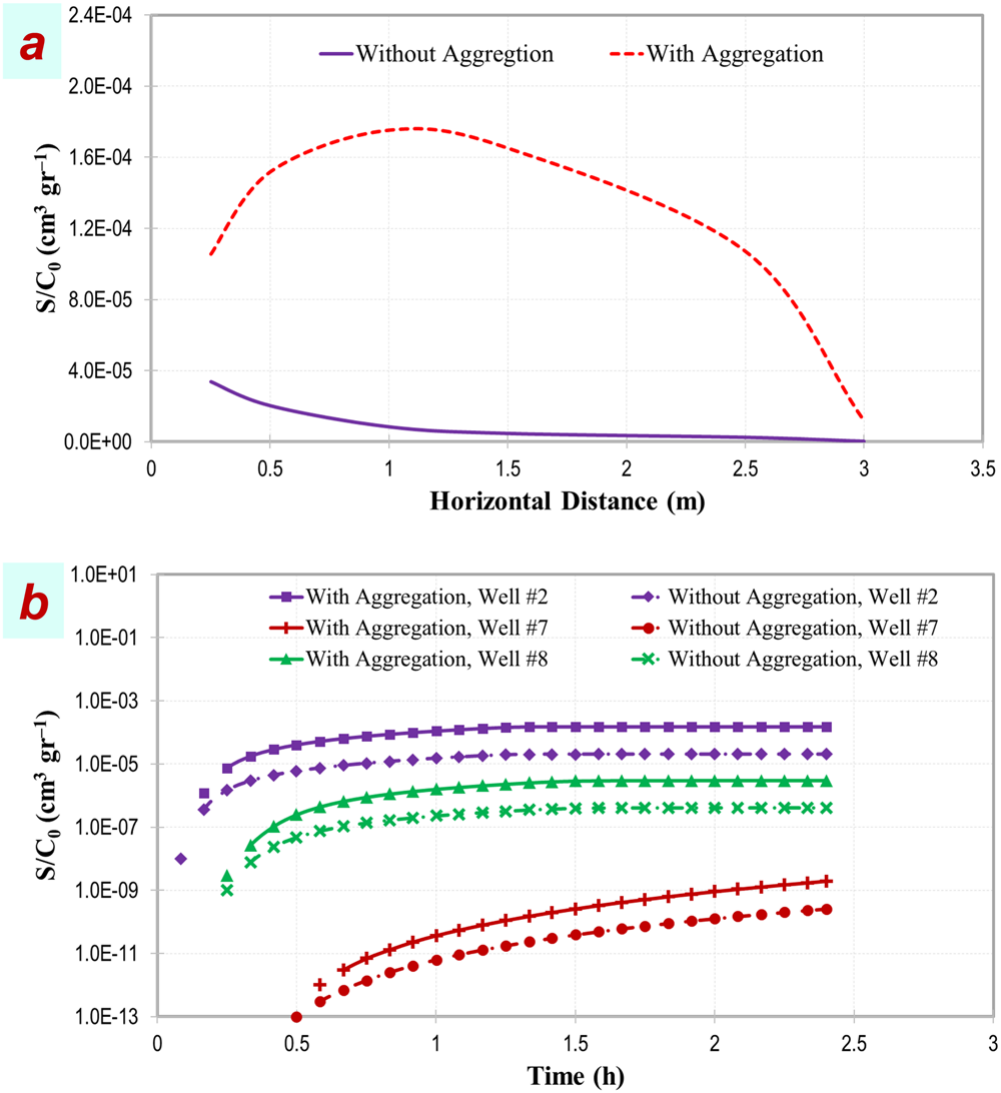
Cumulative residual concentration profiles calculated by particle-volume weighted mean of concentrations across PSDs along the row of observation wells at the centreline of injection-extraction well mainstream (**a**) and its evolution over time at observation wells #2, #7, and #8 without (*α*_*agg*_ = 0) and with (*α*_*agg*_ = 1) incorporating aggregation. Deposition is considered using a size-variable *K*_*att*_ calculated using CFT-DLVO. No acceleration factor has been included.

In the present study, the model-produced RCPs were obtained along the row of observation wells at the centreline of injection-extraction well mainstream without considering acceleration scenarios. Results are shown in Supplementary Fig. S9 as particle size distributed graphs and in Fig, 6a as particle-volume weighted mean of concentrations across PSDs. This investigation demonstrates that without aggregation in the system, RCP peaks at regions close to the injection point (*X* = 0) (Fig. 6a) which is associated with the smallest particles in the PSD (Supplementary Fig. S9). The exponential shape for non-aggregating NP is in agreement with CFT^73^. Based on this model relatively small particles (diameter range 21–190 nm) deposit near the injection point due to their high Brownian diffusion^28^. This is accounted for in the model through the Stokes-Einstein equation considered in the correlation equation of Tufenkji and Elimelech^58^ for determining particle-grain collision efficiencies within the CFT-DLVO approach. Interestingly, with including the aggregation mechanism, the exponential RCP shifts to non-monotonic shape (Fig. 6a) with retention of particles of larger size classes peaking at distances further from the injection point (Supplementary Fig. S9). This is interpreted as initially small particles freshly entering the porous media, interact with each other more significantly than with porous media surfaces and thus they aggregate and grow in size. As a result, their diffusivities decrease^80^ thereby reducing their collision frequencies with porous media in the entrance region. Although a portion of these aggregates are retained in the entrance region, such reduced diffusivity allows part of them to migrate distances within porous media without deposition, until their size grows to the extent that their secondary energy minimum wells calculated for sphere-plate interactions become deep enough or their settling velocity increase sufficiently to induce their retention at lower collision frequencies but with higher efficiency than those of smaller particles^22,30^.

The temporal evolution of normalized cumulative retained particle concentrations based on a particle-volume weighted mean at wells #2, #7, and #8 are shown in Fig. 6b and for different size classes are shown in Supplementary Fig. S10. In line with the above outcomes, the temporal evolution of residual concentration shows that with including aggregation the amount and rate of particle retention increases (Fig. 6b), and the peak of the retained concentration shifts from smaller to larger classes in all three wells (Supplementary Fig. S10). These results suggest why aggregates in larger size classes in BTC-PSD graphs discussed previously did not seem to arrive significantly earlier than intermediate classes even with considering acceleration factor. This is due to retention of grown aggregates following their aggregation hindering the emergence of grown particle masses in BTC-PSD (Supplementary Figs. S5-S8) and causing their appearance in the retained-phase RCP-PSD.

It should be noted that other deposition phenomena such as straining, blocking, and ripening, and other factors such as dynamic effects of pore flow on aggregation process are yet to be incorporated in the model^29,63,73,74,81–85^. Modelling approaches to these factors are currently mostly descriptive rather than predictive^22,62^. Particularly, straining which may be a particle size-dependant phenomenon has been described using either a depth-based or a concentration-based expression, of which none consider the impact of particle size^22,86^. Predictive models taking particle size dynamics into account for such mechanisms require further studies. It should be mentioned that in the range of aggregate sizes reached during aggregation for both suspended and retained aggregates, the straining mechanism may not affect the results of the present study, because as can be seen in Supplementary Figs. S1, S5-S7, the maximum size reached in this study is smaller than 1000 nm yielding a ration of particle diameter to grain diameter (dp/dg) 0.0017 which is the same as the commonly reported critical ration for straining^22,87^. suggesting that straining may not occur for the conditions used in the simulations of the present study.

Overall, the present results provide strong evidence for the hypothesis^22,78,79^ that nonmonotonic RCP appears as a result of concurrent aggregation of NP during transport, and rule out the contribution of site-blocking mechanism in shaping nonmonotonic RCP^88^ as the model presented was able generate such RCPs without considering the blocking mechanism. It should be mentioned that under environmental conditions favourable for aggregation phenomenon particle-particle attachment affinity is deemed to be higher than particle-grain attachment affinity and therefore site blocking which requires higher particle-grain attachment affinity than particle-particle attachment affinity cannot occur^22^. Liang *et al*.^88^ suggest that increasing ionic strength and initial particle concentration bring about more pronounced nonmonotonic RCP. This also agrees with the role of aggregation in producing nonmonotonic RCP, because aggregation intensifies with increasing ionic strength and initial particle concentration^2,30,48^.

The average model runtime of 3-D simulations was 18 ± 4 h on high performance computing (HPC) system. Parallelizing the code using OpenMP capability in Fortran^89^ and using the same number of nodes as the number of size classes did not help reduce the runtime. Although running the modified Fortran code in this study was feasible for forward-modelling of a relatively small 3-D field-scale domain, it can be inefficient to perform the simulation for larger domains and may not be practical when inverse modelling is required too. Therefore, further studies are required to develop more efficient aggregation modelling approaches for combination with continuum models.

## Conclusions

This study considered hypothetical scenarios for size exclusion of NP aggregates revealing that growth in particle size due to aggregation concurrent with their transport within 1-D and 3-D porous media can lead to acceleration in their transport. On a field scale, this acceleration manifested in the form of early arrival of BTCs obtained at observation wells further from the injection point on the mainstream even though the deposition process reduced BTC plateau heights.

This study also sheds light on the underlying cause for non-monotonic RCPs which has been a controversial phenomenon in the literature to date. According to the integrated aggregation-transport model at a 3-D scale developed in this study, exponential RCP with greatest retention close to the injection point is obtained for NP without aggregation being operative, whereas non-monotonic RCP results when aggregation is in operation, inhibiting deposition of a part of individual particles near the injection point by reducing their diffusion and allowing them to migrate up to certain distances where their secondary interaction energy wells or their gravity have increased sufficiently, due to their grown sizes, to retain them.

Overall, NP aggregation can lead to their size exclusion, which is classically identified as early arrival of the BTC. Even without including an acceleration term in the model, aggregation gives rise to a shift in the peak of RCP from regions close to the injection point to further distances, indicating an emergent type of size exclusion that can deliver NP aggregates at greater distances or to less flow-accessible regions in subsurface environments such as semi-permeable confining layers. Although this might be favourable for groundwater remediation operations, this points to the critical risks of offsite migration of NP aggregates to drinking water resources. Therefore, this study highlights the crucial need for accurate simulation and risk analysis of NP implementation strategies within environmental compartments, especially groundwater, before an operation is performed.

## Methods

### Model implementation and modification

The recent release of the Fortran-based public-domain code, MT3D-USGS^47,54^, was modified in the present study to incorporate the FP population balance model equations. Equations (3) and (4) can be solved in the MT3D code assuming conventional distribution coefficient parameter *K*_*dk*_ with values ≤ 0. The first-order irreversible reaction rate term for the mobile phase in the MT3D-USGS code is used to represent ${K}_{at{t}_{k}}$. The subroutines of the MT3D-USGS were modified to incorporate the FP model equations (Eqs 1, 2) as illustrated in the flowcharts of the model algorithms in Supplementary Fig. S12. In doing so, the FP model equations are solved based on number concentration in the most inner loop of the code following a conversion of mass concentration to number concentration for each size class within each time step. The calculated increment in number concentration is then converted back to mass concentration which is exerted on the transport equation solution for each size class within each transport time step. Calculation of the required inputs for the modified MT3D-USGS code, i.e., collision frequencies and transport parameters, was conducted using a MATLAB code as shown in Supplementary Fig. S12. All other model equations are available in the SI and elsewhere^22,48,57,58,61,62^.

### Model parameters

Since currently relatively reliable and comprehensive prediction of model parameters is only possible for attachment rate coefficient, other deposition mechanisms, i.e., straining, ripening, and site blocking, are ignored in the scope of this study which is based on forward-modelling at 3-D scale. The attachment rate coefficient, *K*_*att*_, for each size class was calculated according to the CFT^57,58^ combined with the DLVO theory^59–61^ designated herein as the CFT-DLVO with details available in the SI. The interaction energy forces included in the extended DLVO calculations of the present study were van der Waals attraction, electrostatic repulsion, and Born repulsion after Bradford and Torkzaban^61^. Alternatively, recently developed, ANN-based empirical correlations^62^ were used to calculate *K*_*att*_. Using the latter approach, the detachment rate coefficient, *K*_*det*_, was also incorporated into the model. However, the use of ANN-based empirical correlations is currently limited to the 1-D domain.

Although a variable particle-particle attachment efficiency, *α*_*agg*_, with size, predicted using the DLVO theory^48,90^, was also incorporated into the model, herein a constant *α*_*agg*_ is used for the sake of simplicity since current theories are not able to fully describe aggregation mechanisms in porous media systems due to complex interacting influences of pore tortuosity and the arrival of aggregates from up-gradient pores causing heterogeneous mixing of aggregate populations^29^. Considering *α*_*agg*_ as an unknown constant parameter allows the circumvention of such complex impacts. Model parameters were assumed spatially constant. As such, the calculation of *K*_*att*_ was performed using a constant velocity averaged over the entire model domain (Supplementary Tables S1 and S2).

### Size exclusion consideration

Various approaches have been proposed to model size exclusion as reviewed previously^22^. Among them, one approach is to use a size-variable dispersivity parameter^23,24,91,92^. However, this may not be applicable in the present study due to complexities introduced by the existence of multiple NP transport mechanisms and due to heterogeneous 3-D flow field making it difficult to use a size-dependent dispersivity. Besides, the dispersivity parameter is typically assumed to be dependent only on porous media characteristics such as the scale rather than material properties^22,93,94^. Instead of describing size exculsion using the dipersivity parameter, in the present study, size exclusion was considered by incorporating an “artificial acceleration factor” (a retardation factor ≤ 1)^22,55,56^ via *K*_*d*_ parameter in the MT3D model^54^ with values < 0. It should be noted that this parameter does not represent conventional distribution coefficient in the scope of this study. Two scenarios are considered for producing size exclusion. In the first scenario *K*_*d*_ was assumed constant in terms of aggregate size classes and in the second scenario it was varied linearly with aggregate size (Eq. S3), yielding matched BTC with that of no-*K*_*d*_ for nonaggregating NP dispersions. It should be mentioned that the approach used to investigate size exclusion in the present study, although has already been used in the literature^22,55,56^, might not be a physical representation of the phenomenon but here aims at hypothetical investigation of potential impacts that growth in particle size can have on their transport. Following the conventional approach mentioned above^22,93,94^, it is assumed that the dispersivity parameter is constant with respect to particle size since in the range of aggregate sizes reached in the present study (<1000 nm), theoretical variation^26^ of this parameter with size is negligible.

### Simulation characteristics

The modified MT3D-USGS code was validated against the previous MATLAB code^48^ under a pure aggregation condition. The predictive model performance was assessed against 1-D experimental data selected from Wang *et al*.^49^ in order to be comparable also with the previous study^62^. Such data include HAp NP transport BTCs, the details of which are summarized in Supplementary Table S1.

Three-dimensional simulations were conducted based on field-scale experiments of Johnson *et al*.^31^ on the transport of nanoscale zero valent iron (NZVI) in a confined aquifer. Only experimental domain and flow regime characteristics of that study were considered in the present simulations. The injected particles were assumed to be HAp NP, with the same properties as those of 1-D simulations. All characteristics of 3-D simulations are outlined in Supplementary Table S2 and are described briefly as follows. The model domain is shown in Fig. 1, and space discretization is shown in Supplementary Fig. S13. In brief, the active part of the domain consists of a sand layer confined by silica flour layer at the top and a sandy/clay layer at the bottom which is underlain by a clay layer^31^. Three injection wells and six extraction wells are used to circulate the groundwater in the main confined sand layer. Nanoparticle dispersions are injected only through the middle injection well. The transport of NP is monitored using a row of observation wells at the centreline of the model domain plan (wells #1–6) following Johnson *et al*.^31^, and two more monitoring wells are considered in the simulations, to track the NP transport; one within the lower semi-impermeable sandy/clay layer with permeability *K* = 0.001 cm/s in the direction of the first row of wells (well #7) and another at the top of the main sand layer in the direction of the third row of wells (well #8) as illustrated in Fig. 1. Fifty size classes were considered in the 1-D simulation and 35 classes were considered in the 3-D modelling.

The initial PSD was assumed to be a log-normal distribution after the previous study^48^ yielding a mean initial hydrodynamic diameter, *D*_*H*_, of ~80 nm which is similar to HAp NP dimensions, 20 nm in width and 100 nm in length, reported by Wang *et al*.^49^. The size classes were selected based on a geometric discretization^48^ in a range of 18.2 nm and 2.41 × 10^4^ nm for 1-D simulations and 18.2 nm and 2.67 × 10^3^ for 3-D simulations. In all simulations it was checked that the maximum size is not reached during aggregation. Field-scale simulations were performed on the Chadwick HPC at the University of Liverpool, consisting of single, quad- and dual-core nodes (1.6–2.33 GHz), accessible via Linux operating system.

## Supplementary information

Supplementary Information

## Data Availability

All data including Fortran and MATLAB codes modified or developed in the present study are available upon reasonable request to the author.

## References

[1] Yu, S., Wang, X., Tan, X. & Wang, X. Sorption of radionuclides from aqueous systems onto graphene oxide-based materials: a review. Inorganic Chemistry Frontiers (2015).

[2] Phenrat, T. & Lowry, G. V. *Nanoscale Zerovalent Iron Particles for Environmental Restoration*. (Springer, 2019).

[3] Zhang, T. *et al*. *In situ* remediation of subsurface contamination: Opportunities and challenges for nanotechnology and advanced materials. Environmental Science: Nano (2019).

[4] Kanel SR, Clement TP, Barnett MO, Goltz MN (2011). Nano-Scale Hydroxyapatite: Synthesis, Two-Dimensional Transport Experiments, and Application for Uranium Remediation. Journal of Nanotechnology.

[5] Park, C. M., Wang, D. & Su, C. In *Handbook of Nanomaterials for Industrial Applications* 849–882 (Elsevier, 2018).

[6] Ehtesabi H, Ahadian MM, Taghikhani V, Ghazanfari MH (2013). Enhanced heavy oil recovery in sandstone cores using tio2 nanofluids. Energy & Fuels.

[7] Hashemi R, Nassar NN, Pereira Almao P (2013). Enhanced Heavy Oil Recovery by *in Situ* Prepared Ultradispersed Multimetallic Nanoparticles: A Study of Hot Fluid Flooding for Athabasca Bitumen Recovery. Energy & Fuels.

[8] Abdelfatah E, Kang K, Pournik M, Shiau BJB, Harwell J (2017). Mechanistic study of nanoparticles deposition and release in porous media. Journal of Petroleum Science and Engineering.

[9] Wang D, Jin Y, Jaisi DP (2015). Effect of Size-Selective Retention on the Cotransport of Hydroxyapatite and Goethite Nanoparticles in Saturated Porous Media. Environmental science & technology.

[10] Kah, M., Kookana, R. S., Gogos, A. & Bucheli, T. D. A critical evaluation of nanopesticides and nanofertilizers against their conventional analogues. *Nature nanotechnology*, **1** (2018). 10.1038/s41565-018-0131-1 29736032

[11] Dimkpa, C. O. & Bindraban, P. S. Nanofertilizers: New products for the industry? *J. Agric. Food. Chem*. (2017). 10.1021/acs.jafc.7b02150 28535672

[12] Keller AA, Lazareva A (2013). Predicted releases of engineered nanomaterials: from global to regional to local. Environmental Science & Technology Letters.

[13] Keller AA, McFerran S, Lazareva A, Suh S (2013). Global life cycle releases of engineered nanomaterials. J. Nanopart. Res..

[14] Royal-Society. 116 (Royal Society, Royal Academy of Engineering (Great Britain), 2004).

[15] Patil SS, Shedbalkar UU, Truskewycz A, Chopade BA, Ball AS (2016). Nanoparticles for environmental clean-up: a review of potential risks and emerging solutions. Environmental Technology & Innovation.

[16] Kersting AB (1999). Migration of plutonium in ground water at the Nevada Test Site. Nature.

[17] McCarthy JF, Zachara JM (1989). Subsurface transport of contaminants. Environmental Science & Technology.

[18] Xie, J. et al. Insights into transport velocity of colloid-associated plutonium relative to tritium in porous media. *Scientific reports* **4** (2014). 10.1038/srep05037 PMC4052758 24849695

[19] Kheirabadi M, Niksokhan MH, Omidvar B (2017). Colloid-Associated Groundwater Contaminant Transport in Homogeneous Saturated Porous Media: Mathematical and Numerical Modeling. Environmental Modeling & Assessment.

[20] Wen T (2014). Efficient capture of strontium from aqueous solutions using graphene oxide–hydroxyapatite nanocomposites. Dalton Transactions.

[21] Kamrani S, Rezaei M, Kord M, Baalousha M (2018). Co-transport and remobilization of Cu and Pb in quartz column by carbon dots. Sci. Total Environ..

[22] Babakhani P, Bridge J, Doong R-a, Phenrat T (2017). Continuum-based models and concepts for the transport of nanoparticles in saturated porous media: A state-of-the-science review. Adv. Colloid Interface Sci..

[23] Auset, M. & Keller, A. A. Pore‐scale processes that control dispersion of colloids in saturated porous media. *Water Resour. Res*. **40** (2004).

[24] Keller, A. A., Sirivithayapakorn, S. & Chrysikopoulos, C. V. Early breakthrough of colloids and bacteriophage MS2 in a water‐saturated sand column. *Water Resour. Res*. **40** (2004).

[25] Prieve DC, Hoysan PM (1978). Role of colloidal forces in hydrodynamic chromatography. J. Colloid Interface Sci..

[26] James SC, Chrysikopoulos CV (2003). Effective velocity and effective dispersion coefficient for finite-sized particles flowing in a uniform fracture. J. Colloid Interface Sci..

[27] Wang Y (2013). Effect of surface coating composition on quantum dot mobility in porous media. J. Nanopart. Res..

[28] Molnar, I. L., Johnson, W. P., Gerhard, J. I., Willson, C. S. & O’Carroll, D. M. Predicting colloid transport through saturated porous media: A critical review. Water Resour. Res. (2015).

[29] Babakhani, P., Bridge, J., Phenrat, T., Doong, R.-a. & Whittle, K. Aggregation and sedimentation of shattered graphene oxide nanoparticles in dynamic environments: a solid-body rotational approach. Environmental Science: Nano 10.1039/C8EN00443A (2018).

[30] Phenrat T (2009). Particle size distribution, concentration, and magnetic attraction affect transport of polymer-modified Fe0 nanoparticles in sand columns. Environ. Sci. Technol..

[31] Johnson RL (2013). Field-Scale Transport and Transformation of Carboxymethylcellulose-Stabilized Nano Zero-Valent Iron. Environmental science & technology.

[32] El Badawy AM, Aly Hassan A, Scheckel KG, Suidan MT, Tolaymat TM (2013). Key factors controlling the transport of silver nanoparticles in porous media. Environmental science & technology.

[33] Fan W (2015). Transport of graphene oxide in saturated porous media: Effect of cation composition in mixed Na–Ca electrolyte systems. Sci. Total Environ..

[34] Mitzel MR, Tufenkji N (2014). Transport of industrial PVP-stabilized silver nanoparticles in saturated quartz sand coated with Pseudomonas aeruginosa PAO1 biofilm of variable age. Environmental science & technology.

[35] Sagee O, Dror I, Berkowitz B (2012). Transport of silver nanoparticles (AgNPs) in soil. Chemosphere.

[36] Cornelis G, Pang L, Doolette C, Kirby JK, McLaughlin MJ (2013). Transport of silver nanoparticles in saturated columns of natural soils. Sci. Total Environ..

[37] Solovitch N (2010). Concurrent aggregation and deposition of TiO2 nanoparticles in a sandy porous media. Environmental science & technology.

[38] Laumann S, Micić V, Lowry GV, Hofmann T (2013). Carbonate minerals in porous media decrease mobility of polyacrylic acid modified zero-valent iron nanoparticles used for groundwater remediation. Environ. Pollut..

[39] Cohen, M. & Weisbrod, N. Field scale mobility and transport manipulation of carbon-supported nanoscale zero-valent iron (nZVI) in fractured media. *Environmental science & technology* (2018). 10.1021/acs.est.8b01226 29900735

[40] Troester M, Brauch H-J, Hofmann T (2016). Vulnerability of drinking water supplies to engineered nanoparticles. Water Res..

[41] Dale AL (2015). Modeling Nanomaterial Environmental Fate in Aquatic Systems. Environmental Science & Technology.

[42] Chatterjee J, Gupta SK (2009). An agglomeration-based model for colloid filtration. Environmental Science & Technology.

[43] Raychoudhury T, Tufenkji N, Ghoshal S (2012). Aggregation and deposition kinetics of carboxymethyl cellulose-modified zero-valent iron nanoparticles in porous media. Water Res..

[44] Taghavy A, Pennell KD, Abriola LM (2015). Modeling coupled nanoparticle aggregation and transport in porous media: A Lagrangian approach. J. Contam. Hydrol..

[45] Babakhani Peyman, Fagerlund Fritjof, Shamsai Abolfazl, Lowry Gregory V., Phenrat Tanapon (2015). Modified MODFLOW-based model for simulating the agglomeration and transport of polymer-modified Fe0 nanoparticles in saturated porous media. Environmental Science and Pollution Research.

[46] Phenrat T, Kim H-J, Fagerlund F, Illangasekare T, Lowry GV (2010). Empirical correlations to estimate agglomerate size and deposition during injection of a polyelectrolyte-modified Fe0 nanoparticle at high particle concentration in saturated sand. J. Contam. Hydrol..

[47] Bedekar, V., Morway, E. D., Langevin, C. D. & Tonkin, M. J. MT3D-USGS version 1: A US Geological Survey release of MT3DMS updated with new and expanded transport capabilities for use with MODFLOW. Report No. 2328-7055, (US Geological Survey, 2016).

[48] Babakhani Peyman, Doong Ruey-an, Bridge Jonathan (2018). Significance of Early and Late Stages of Coupled Aggregation and Sedimentation in the Fate of Nanoparticles: Measurement and Modeling. Environmental Science & Technology.

[49] Wang D (2011). Transport behavior of humic acid-modified nano-hydroxyapatite in saturated packed column: effects of Cu, ionic strength, and ionic composition. J. Colloid Interface Sci..

[50] Vermeul VR (2014). An injectable apatite permeable reactive barrier for *in situ* 90Sr immobilization. Groundwater Monitoring & Remediation.

[51] Handley-Sidhu S (2011). Uptake of Sr2+ and Co2+ into biogenic hydroxyapatite: implications for biomineral ion exchange synthesis. Environmental science & technology.

[52] Kumar S, Ramkrishna D (1996). On the solution of population balance equations by discretization—I. A fixed pivot technique. Chem. Eng. Sci..

[53] Atmuri AK, Henson MA, Bhatia SR (2013). A population balance equation model to predict regimes of controlled nanoparticle aggregation. Colloids and Surfaces A: Physicochemical and Engineering Aspects.

[54] Zheng, C. & Wang, P. P. A modular three-dimensional multi-species transport model for simulation of advection, dispersion and chemical reactions of contaminants in groundwater systems; documentation and user’s guide. US Army Engineer Research and Development Center Contract Report SERDP-99-1, Vicksburg, Mississippi, USA (1999).

[55] Sirivithayapakorn, S. & Keller, A. Transport of colloids in saturated porous media: A pore‐scale observation of the size exclusion effect and colloid acceleration. *Water Resour. Res*. **39** (2003).

[56] Van Genuchten, M. T. Non-equilibrium transport parameters from miscible displacement experiments (1981).

[57] Yao K-M, Habibian MT, O’Melia CR (1971). Water and waste water filtration. Concepts and applications. Environmental Science & Technology.

[58] Tufenkji N, Elimelech M (2004). Correlation equation for predicting single-collector efficiency in physicochemical filtration in saturated porous media. Environmental Science & Technology.

[59] Adamczyk Z, Weroński P (1999). Application of the DLVO theory for particle deposition problems. Adv. Colloid Interface Sci..

[60] Van Oss CJ, Giese RF, Costanzo PM (1990). DLVO and non-DLVO interactions in hectorite. Clays Clay Miner..

[61] Bradford SA, Torkzaban S (2015). Determining parameters and mechanisms of colloid retention and release in porous media. Langmuir.

[62] Babakhani P, Bridge J, Doong R-A, Phenrat T (2017). Parameterization and prediction of nanoparticle transport in porous media: A reanalysis using artificial neural network. Water Resour. Res..

[63] Saiers JE, Hornberger GM, Liang L (1994). First–and second–order kinetics approaches for modeling the transport of colloidal particles in porous media. Water Resour. Res..

[64] Phenrat T (2010). Estimating Attachment of Nano- and Submicrometer-particles Coated with Organic Macromolecules in Porous Media: Development of an Empirical Model. Environmental Science & Technology.

[65] Torkzaban S, Wan J, Tokunaga TK, Bradford SA (2012). Impacts of bridging complexation on the transport of surface-modified nanoparticles in saturated sand. J. Contam. Hydrol..

[66] Ives KJ (1970). Rapid filtration. Water Res..

[67] Herzig JP, Leclerc DM, Goff PL (1970). Flow of Suspensions through Porous Media—Application to Deep Filtration. Industrial & Engineering Chemistry.

[68] Phenrat T (2010). Transport and deposition of polymer-modified Fe0 nanoparticles in 2-D heterogeneous porous media: Effects of particle concentration, Fe0 content, and coatings. Environmental Science & Technology.

[69] Afshinnia K, Gibson I, Merrifield R, Baalousha M (2016). The concentration-dependent aggregation of Ag NPs induced by cystine. Sci. Total Environ..

[70] Friedlander SK (1960). Similarity considerations for the particle-size spectrum of a coagulating, sedimenting aerosol. Journal of Meteorology.

[71] Jeffrey DJ (1981). Quasi-stationary approximations for the size distribution of aerosols. Journal of the Atmospheric Sciences.

[72] Hunt JR (1982). Self-similar particle-size distributions during coagulation: theory and experimental verification. J. Fluid Mech..

[73] Bradford SA, Bettahar M (2006). Concentration dependent transport of colloids in saturated porous media. J. Contam. Hydrol..

[74] Bradford SA, Simunek J, Bettahar M, van Genuchten MT, Yates SR (2003). Modeling colloid attachment, straining, and exclusion in saturated porous media. Environmental science & technology.

[75] Li Y, Wang Y, Pennell KD, Abriola LM (2008). Investigation of the transport and deposition of fullerene (C60) nanoparticles in quartz sands under varying flow conditions. Environmental science & technology.

[76] Wang Y, Becker MD, Colvin VL, Abriola LM, Pennell KD (2014). Influence of Residual Polymer on Nanoparticle Deposition in Porous Media. Environmental Science & Technology.

[77] Bradford, S. A., Simunek, J. & Walker, S. L. Transport and straining of *E. coli* O157: H7 in saturated porous media. *Water Resour. Res*. **42** (2006). 10.1016/j.watres.2009.09.027 19853881

[78] Chen G, Liu X, Su C (2012). Distinct effects of humic acid on transport and retention of tio2 rutile nanoparticles in saturated sand columns. Environmental Science & Technology.

[79] Chen G, Liu X, Su C (2011). Transport and retention of TiO2 rutile nanoparticles in saturated porous media under low-ionic-strength conditions: measurements and mechanisms. Langmuir.

[80] Lin S, Wiesner MR (2012). Deposition of Aggregated Nanoparticles· A Theoretical and Experimental Study on the Effect of Aggregation State on the Affinity between Nanoparticles and a Collector Surface. Environmental science & technology.

[81] Xu, S., Gao, B. & Saiers, J. E. Straining of colloidal particles in saturated porous media. *Water Resour. Res*. **42** (2006).

[82] Wang D, Su C, Liu C, Zhou D (2014). Transport of fluorescently labeled hydroxyapatite nanoparticles in saturated granular media at environmentally relevant concentrations of surfactants. Colloids and Surfaces A: Physicochemical and Engineering Aspects.

[83] Tosco T, Sethi R (2010). Transport of non-Newtonian suspensions of highly concentrated micro-and nanoscale iron particles in porous media: a modeling approach. Environmental Science & Technology.

[84] Hosseini SM, Tosco T (2013). Transport and retention of high concentrated nano-Fe/Cu particles through highly flow-rated packed sand column. Water Res..

[85] Shen, C., Huang, Y., Li, B. & Jin, Y. Effects of solution chemistry on straining of colloids in porous media under unfavorable conditions. *Water Resour. Res*. **44** (2008).

[86] Johnson WP, Ma H, Pazmino E (2011). Straining credibility: a general comment regarding common arguments used to infer straining as the mechanism of colloid retention in porous media. Environmental science & technology.

[87] Bradford SA, Yates SR, Bettahar M, Simunek J (2002). Physical factors affecting the transport and fate of colloids in saturated porous media. Water Resour. Res..

[88] Liang Y, Bradford SA, Simunek J, Vereecken H, Klumpp E (2013). Sensitivity of the transport and retention of stabilized silver nanoparticles to physicochemical factors. Water Res..

[89] Hermanns, M. *Parallel programming in Fortran 95 using OpenMP*. Universidad Politecnica de Madrid, Spain (2002).

[90] Zhang, W. In *Nanomaterial Impacts on Cell Biology and Medicine* (ed. Yongsheng Chen David G. Capco) Ch. 2, 19–43 (Springer, 2014).

[91] Grolimund Daniel, Elimelech Menachem, Borkovec Michal, Barmettler Kurt, Kretzschmar Ruben, Sticher Hans (1998). Transport of in Situ Mobilized Colloidal Particles in Packed Soil Columns. Environmental Science & Technology.

[92] Chrysikopoulos CV, Katzourakis VE (2015). Colloid particle size‐dependent dispersivity. Water Resour. Res..

[93] Fried, J. J. & Combarnous, M. A. In *Advances in hydroscience* Vol. 7 169–282 (Elsevier, 1971).

[94] Ginn TR (2000). Comment on “Stochastic analysis of virus transport in aquifers,” by Linda L. Campbell Rehmann, Claire Welty, and Ronald W. Harvey. Water Resour. Res..

