# Supplementary material for: The impact of nanoparticle aggregation on their size exclusion during transport in porous media: One- and three-dimensional modelling investigations
Source: Sci Rep. 2019 Oct 1;9:14071. doi: 10.1038/s41598-019-50493-6 (PMC6773746; doi:10.1038/s41598-019-50493-6)
Supplement: Supplementary file 1 — Supplementary Information [file 41598_2019_50493_MOESM1_ESM.pdf]

Supplementary Information for "The impact of nanoparticle aggregation on their size exclusion during transport in porous media: One- and three-dimensional modelling investigations"

Peyman Babakhani[*]

School of Engineering, University of Liverpool, Liverpool, Merseyside L69 3GH, UK

[*]Corresponding author:

p.babakhani@leeds.ac.uk

T +44(0)7913000434

Number of pages: 26

Number of figures: 13

Number of tables: 3

Other equations of the model.

Detachment can be considered using an alternative approach in which the attached particle concentration can also be tracked but the K_d parameter used to consider acceleration factor in Eqs. (3) and (4) is not included anymore:[1,2]

$$\varepsilon \frac{\partial C_k}{\partial \mathrm{t}} + \rho_b \frac{\partial S_k}{\partial \mathrm{t}} = \frac{\partial}{\partial x_L}\left(\varepsilon D_{L,T} \frac{\partial C_k}{\partial x_T}\right) - \frac{\partial(\varepsilon V_L C_k)}{\partial x_L} \qquad \text{(S1)}$$

$$\rho_b \frac{\partial S_k}{\partial \mathrm{t}} = \varepsilon K_{att_k} C_k - \rho_b K_{det_k} S_k \qquad \text{(S2)}$$

where S_k is the mass concentration of deposited phase particles of size class k [MM^{-3}] and K_{det_k} is detachment rate coefficient for size class k [T^{-1}]. These equations can be solved using the MT3D code when a non-equilibrium sorption model is considered. In doing so, model parameters for each size class can be converted as: $K_{att_k} = \beta_k / \varepsilon$ and $K_{det_k} = \beta_k / (\rho_b K_{d_k})$. In MT3D notation[3], β_k is the first-order mass transfer rate between the mobile and retained phases for species k. It should be noted that detachment can be excluded from this model formulation in the MT3D code by assigning a large value to K_{d_k}.[4,5]

In scenarios including size exclusion, a variable K_d was assumed to vary with size according to the following linear equation:

$$K_{d_k} = K_{d_{max}} - \frac{a_k}{a_{max}}\left(K_{d_{max}} - K_{d_{min}}\right) \qquad \text{(S3)}$$

where K_{d_k}, $K_{d_{max}}$, and $K_{d_{min}}$ are K_d values for a given size class k, for size class with smallest particles, and for size class with largest aggregates, respectively; a_k is the radius of size class k and a_{max} is the radius of largest aggregate size class.

The collision kernel for environmental colloids is commonly given as the sum of three mechanisms: perikinetic collisions (Brownian), orthokinetic collisions (shear-induced aggregation under fluid motion), and differential settling (collection of smaller aggregates by the larger ones during sedimentation).[6] Expressing collision frequencies based on the volume (or mass) of aggregates as a representative variable,[7,8] using fractal dimension relationships and considering permeability drag effects[9] the following relationships yield:

$$\beta_{Prik_{i,j}} = \frac{2k_bT}{3\mu}\left(v_i^{\left(\frac{1}{D_f}\right)} + v_j^{\left(\frac{1}{D_f}\right)}\right)\left(\frac{1}{\Omega_i}v_i^{-\left(\frac{1}{D_f}\right)} + \frac{1}{\Omega_j}v_j^{-\left(\frac{1}{D_f}\right)}\right) \quad \text{(S4)}$$

$$\beta_{Orth_{i,j}} = \frac{G}{\pi}v_0^{\left(1-\frac{3}{D_f}\right)}\left((\eta_{c_i}{}^{\left(\frac{1}{2}\right)}\, v_i^{\left(\frac{1}{D_f}\right)} + \eta_{c_j}{}^{\left(\frac{1}{2}\right)}\, v_j^{\left(\frac{1}{D_f}\right)}\right)^3 \quad \text{(S5)}$$

$$\beta_{Diff_{i,j}} = \frac{3}{2}\left(\frac{\pi}{6}\right)^{\frac{1}{3}} v_0^{\left(\frac{2}{3}-\frac{2}{D_f}\right)}\left((\eta_{c_i}{}^{\left(\frac{1}{2}\right)}\, v_i^{\left(\frac{1}{D_f}\right)} + \eta_{c_j}{}^{\left(\frac{1}{2}\right)}\, v_j^{\left(\frac{1}{D_f}\right)}\right)^2 \left|U_i - U_j\right| \quad \text{(S6)}$$

where μ is the dynamic viscosity of the suspending fluid, k_B is the Boltzmann constant (1.381×10^{-23}), T is the temperature (°K), v_i and v_j are representative solid volumes of each aggregate in size classes i and j, D_f is the fractal dimension of aggregates, G is the volume-averaged fluid velocity gradient or shear rate (herein it was assumed as zero), v_0 is the volume of individual primary particles or smallest size class, g is the acceleration due to gravity, and U_i and U_j are the settling velocities of agglomerates at respective size classes, Ω_i and Ω_j are the drag coefficient correction factor of aggregates at respective size classes, defined as the ratio of drag force exerted on a permeable aggregate to drag force exerted on an impervious aggregate with the same size,[10,11] and η_{c_i} and η_{c_j} are the fluid collection efficiency of aggregates at respective size, defined as the ratio of flow through an aggregate to total flow approaching the aggregate.[11,12]

The superposition of the three collision frequencies gives the total rate of collisions, $\beta(i,j)$:

$$\beta_{i,j} = \beta_{Prik_{i,j}} + \beta_{Orth_{i,j}} + \beta_{Diff_{i,j}} \quad \text{(S7)}$$

The drag coefficient correction factor, Ω, is given as:[10,11,13,14]

$$\Omega = \frac{2\xi^2\left(1 - \frac{\tanh(\xi)}{\xi}\right)}{2\xi^2 + 3\left(1 - \frac{\tanh(\xi)}{\xi}\right)} \quad \text{(S8)}$$

where ξ is the non-dimensional permeability of the porous aggregate given as:

$$\xi = \frac{a}{\kappa^{1/2}} \quad \text{(S9)}$$

where κ is permeability [L^2] as described later.

The fluid collection efficiency, η_c, can be determined from the Brinkman equation:[11,12]

$$\eta_c = 1 - \frac{d_c}{\xi} - \frac{c_c}{\xi^3} \tag{S10}$$

where

$$d_c = \frac{3}{J}\xi^3\left(1 - \frac{\tanh(\xi)}{\xi}\right) \tag{S11}$$

$$c_c = -\frac{1}{J}\left(\xi^5 + 6\xi^3 - \frac{\tanh(\xi)}{\xi}(3\xi^5 + 6\xi^3)\right) \tag{S12}$$

$$J = 2\xi^2 + 3 - 3\frac{\tanh(\xi)}{\xi} \tag{S13}$$

Babakhani, et al.[15] examined four different approaches to the settling velocity of HAp NP and found that a power-law formulation is the best model:

$$\frac{U}{U_0} = \left(\frac{d_k}{d_0}\right)^{D_f - 1}. \tag{S14}$$

where d_0 and d_k are diameters of the smallest and the given size class k, respectively and U_0 is the settling velocity for primary particles [L T^{-1}], which can be determined from the Stokes' equation:[16]

$$U_0 = \frac{2g}{9\mu}(\rho_0 - \rho_w){a_0}^2 \tag{S15}$$

where ρ_0 is density of primary particles [ML^{-3}], and ρ_w is density of fluid [ML^{-3}].

Babakhani et al.[15] also investigated three different approaches to calculate the permeability within the collision frequency formulations for HAp NP and found Brinkman's model is the best:[12]

$$\kappa = \frac{(a_0)^2}{18}\left(3 + \frac{3}{(1-\varphi)} - \left(\frac{8}{(1-\varphi)} - 3\right)^{\frac{1}{3}}\right) \tag{S16}$$

where a_0 is the radius of primary particles and φ is porosity given as:[12,17,18]

$$\varphi = 1 - \left(\frac{d_k}{d_0}\right)^{D_f - 3} \tag{S17}$$

The representative volume of aggregates in each size class, v_k, can be calculated as:

$$v_k = \frac{\pi}{6}(d_0)^{3-D_f}(d_k)^{D_f} \tag{S18}$$

The mass of each aggregate can then be calculated as:[18]

$$m_k = \rho_0 v_k \tag{S19}$$

where ρ_0 is the density of the particles and m_k is the mass of each aggregate in size class k. The conversion between the mass concentration C_k, and the number concentration n_k, is then performed via:

$$C_k = m_k \times n_k \tag{S20}$$

Calculation of attachment rate coefficient

Two approaches are used to calculate the attachment rate coefficient, K_{att_k}: First using a combination of colloid filtration theory (CFT)[19,20] and Derjaguin, Landau, Verwey, and Overbeek (DLVO) theory[21] (CFT-DLVO); Second based on ANN-based empirical correlations developed by Babakhani et al.[22]

Attachment rate coefficient can be calculated using CFT-DLVO as follows:[20]

$$K_{att_k} = \frac{3(1-\varepsilon)}{2d_{50}} \alpha_{pc_k} \eta_{0_k} V_{ave} \tag{S21}$$

where d_{50} is the median porous media grain size [L], α_{pc_k} is the attachment efficiency for interactions between particles in size class k and collectors (porous media grains), η_{0_k} is the collision or contact efficiency of particles in size class k and collectors, and V_{ave} is the pore water velocity [L T^{-1}] which in the present study is averaged over the spatial domain of the model. Collision efficiency, which is related to the flux of particles approaching the surface of a porous media grain, can be calculated using the well-established correlation equation of Tufenkji and Elimelech.[20] Despite the existence of several empirical correlations developed for calculating α_{pc_k}, [23,24] here a theoretical approach based on the extended DLVO theory is used following Bradford and Torkzaban:[21]

$$\alpha_{pc} = \varepsilon_1 \gamma_1 + \varepsilon_2 \gamma_2 \tag{S22}$$

where ε_1 and ε_1 are the probabilities of colloid interaction with the primary and secondary energy minima of DLVO, respectively, and $\gamma_1$and γ_2 are parameters representing the role of applied hydrodynamic torque (T_H) and adhesive torque (T_A) in the above equation—equal to one when $T_H \leq T_A$ and zero when $T_H > T_A$. Here since it is assumed that deposition is irreversible when CFT-DLVO approach is used, γ_1 and γ_2 are assumed to be one. Based on energy balance, ε_j (j = 1 or 2) can be calculated:[21]

$$\varepsilon_j = \int_{l_A}^{l_B} \frac{2\sqrt{\Phi}}{\pi} \exp(-\Phi)\, d\Phi = \left(\mathrm{erf}\left(\sqrt{l_B}\right) - \sqrt{\frac{4l_B}{\pi}}\, exp(-l_B)\right) - \left(erf\left(\sqrt{l_A}\right) - \sqrt{\frac{4l_A}{\pi}}\, exp(-l_A)\right) \quad \text{(S23)}$$

where l_A and l_B are the lower and the upper integration limits that will be discussed later, and Φ is the mean value of the dimensionless total interaction energy determined from extended DLVO. The total interaction energy [Kg m^2 S^{-2}], Φ_d, is calculated as a sum of three interaction energies, i.e., van der Waals attraction, Φ^{vdW}, electrostatic repulsion, Φ^{el}, and Born repulsion, Φ^{Born}:

$$\Phi(h) = \Phi^{vdW}(h) + \Phi^{el}(h) + \Phi^{Born}(h) \quad \text{(S24)}$$

where h is the separation distance [L] between the colloid and the solid-water interface.

The sphere-plate van der Waals interaction energy can be calculated using the expression of Gregory:[25]

$$\Phi^{vdW}(h) = -\frac{A_{H123} a_k}{6h}\left[1 + \frac{14h}{\lambda}\right]^{-1} \quad \text{(S25)}$$

where A_{H123} is the combined Hamaker constant [ML^{2}T^{-2}] for the particle-water-surface system, and λ is a characteristic wavelength assumed equal to 100 nm.[21] The combined Hamaker constant for hydroxyapatite NP interacting with water-quartz interface is 2.94×10^{-21} J.[26]

The electrostatic repulsion energy for a sphere-plate interaction is given as:[27]

$$\Phi^{el}(h) = \pi\varepsilon_m\varepsilon_0 a_k \left\{2\varphi_1\varphi_2 ln\left[\frac{1 + exp(-\kappa_{Deb}h)}{1 - exp(-\kappa_{Deb}h)}\right] + ({\varphi_1}^2 + {\varphi_2}^2)\, ln[1 - exp(-2\kappa_{Deb}h)]\right\} \quad \text{(S26)}$$

where ε_0 is the permittivity of a vacuum which is taken as 8.854× 10^{-12} C/(V·m), ε_m is the dielectric constant of the medium [–] taken as 78.5, φ_1 and φ_2 are the surface potentials (mV) for the porous media grain and the interacting particle, respectively. Although φ_1 and φ_2 are usually assumed approximately equal to the zeta potential of the two surfaces,[28] in a more accurate way they the can be determined from the following equation:[29-31]

$$\varphi = \zeta\left(1 + \frac{h_s}{a}\right)\exp(\kappa_{Deb} h_s) \tag{S27}$$

where ζ is the zeta potential (V), h_s is the distance between the surface of the charged particles and the slipping plane— usually taken as 5 $\dot{A}$, κ_{Deb} is the inverse of the Debye length (nm) given as:

$$\kappa_{Deb}{}^{-1} = \sqrt{\frac{\varepsilon_m \varepsilon_0 k_B T}{2 N_A I e^2}} \tag{S28}$$

where N_A is Avogadro's number (6.02 × 10^{23} mol^{-1}), e is unit charge, 1.602 × 10^{-19} C, and I is the ionic strength (M), ($I = 0.5 \sum c_i {Z_i}^2$, where c_i is the molar concentration of each ionic species, i, in the solution, and Z_i is the valency of the that ion).

Born repulsion can be calculated as follows:[21]

$$\Phi^{Born}(h) = -\frac{A_{H123}{\sigma_c}^6}{7560}\left[\frac{8a_k + h}{(2a_k + 7)^7} + \frac{6a_k - h}{h^7}\right] \tag{S29}$$

where σ_c is the collision diameter assumed as 0.26 nm in order to achieve a primary minimum depth at 0.157 nm, a commonly accepted distance of closest approach, following Bradford and Torkzaban.[21] The values of l_A and l_B in Eq. (S23) are determined as described in detail by Bradford and Torkzaban.[21] In brief, five types of the interaction energy profile are considered, ranging from favourable to unfavourable interaction condition evaluated using Φ_{1min}, Φ_{2min}, and Φ_{max} standing for primary minimum, secondary minimum, and the energy barrier in the total interaction energy profile as shown in Fig. S11. Based on these classes and ignoring the detachment, the values of l_A and l_B can be determined from Table S3.[21]

In the alternative approach, i.e., calculation of the attachment rate coefficient according to the ANN-based empirical correlations,[22] the empirical matrices of neural network were employed in the MATLAB code developed in the present study. The code used these networks to calculate both attachment and detachment rate coefficients for each size class using 20 experimental

characteristics listed in Table S1. It should be note that these empirical networks were already exerted in an MS Excel spreadsheet and were made available online as electronic supporting information of Babakhani et al.[22] All Fortran and MATLAB codes developed in the present study are available upon reasonable request to the author.

Tables and Figures:

Table S1. Parameters used in 1-D simulations following previous studies.[22,32]

Parameter	50 mM KCl dataset	0.5 mM $CaCl_2$ dataset
**Free polymer concentration (mg/L)**	10, Humic acid	10, Humic acid
**NP zeta potential (mV)**	-28.8	-31.4
**NP average diameter (nm)**	100	100
**NP density (g/cm3)**	3.16	3.2
**NP Concentration (mg/L)**	200	200
**Column diameter (cm)**	2.6	2.6
**Column length (cm)**	20.2	20.2
**Heterogeneity (%)**	15	15
**Grain size, d (mm)**	0.6	0.6
**Grain Zeta potential (mV)**	-40.2	-34.4
**Porosity**	0.39	0.39
**Pore water velocity (cm/min)**	1.12	1.11
**Dispersivity (cm)**	4.8×10^{-3}	6.6×10^{-3}
**pH**	5.7	5.7
**Electrolyte concentration (mM)**	50	0.5
**Number of PVs**	3.75	3.75
**Particle aspect ratio**	5	5
**Adsorbed coating concentration (mg/L)**	0	0
**Saturation magnetization (kA/m)**	0	0
**IEP pH**	6.7	6.7
**PSD size range (nm)**	18.2 to 2.41×10^{4}	18.2 to 2.41×10^{4}
**Number of size classes**	50	50
**Constant K_d (m^3/kg)**	-5.5×10^{-5}	-5.5×10^{-5}
**Variable K_d range (m^3/kg)**	-1.4×10^{-6} to -1.8×10^{-3}	-1.4×10^{-6} to -1.8×10^{-3}
**Soil bulk density (kg/m^3)**	1590	1590
**Dispersivity (cm)**	0.048	0.048
**Hamaker constant (A_{H123}) (J)**	2.94×10^{-21}	2.94×10^{-21}
**Fractal dimension**	2.0	2.0

Table S2. Parameters used in 3-D simulations.[32,33]

Parameter	Assumed Values
**NP zeta potential (mV)**	-28.8
**NP average diameter (nm)**	100
**NP density (g/cm3)**	3.16
**NP Concentration (mg/L)**	900 and 4500
**Test dimensions (m)**	3.6(L)×3(W)×2.4(H)
**Grain size, d (mm)**	0.6
**Grain Zeta potential (mV)**	-40.2
**Porosity**	0.35
**Pore water velocity (averaged) (cm/min)**	2.4
**Dispersivity (cm)**	1
**Ionic strength (mM)**	50
**Injection duration (min)**	~67
**PSD size range (nm)**	18.2 to 2.67×10^{3}
**Number of size classes**	35
**Constant K_d (m^3/kg)**	-5.5×10^{-5}
**Variable K_d range (m^3/kg)**	-1.4×10^{-6} to -2.0×10^{-4}
**Soil bulk density (kg/m^3)**	1400
**Longitudinal dispersivity (cm)**	1
**Horizontal to longitudinal dispersivity**	1/10
**Vertical to longitudinal dispersivity**	1/100
**Hamaker constant (A_{H123}) (J)**	2.94×10^{-21}
**Fractal dimension**	2.0

Table S3. Lower (l_A) and upper (l_B) integration limits in calculation of the probabilities of colloid interaction with the primary (ε_1) and secondary (ε_1) minima of DLVO profile based on five categories of interaction energy profile illustrated in Fig. S11.

**Category No.**	**ε1**	**ε1**	**ε2**	**ε2**
	$\boldsymbol{l_A}$	$\boldsymbol{l_B}$	$\boldsymbol{l_A}$	$\boldsymbol{l_B}$
**1**	0	$\lvert\boldsymbol{\Phi_{1min}}\rvert$	NA	NA
**2**	$\lvert\boldsymbol{\Phi_{2min} - \Phi_{max}}\rvert$	$\lvert\boldsymbol{\Phi_{1min} - \Phi_{max}}\rvert$	0	$\lvert\boldsymbol{\Phi_{2min} - \Phi_{max}}\rvert$
**3**	$\lvert\boldsymbol{\Phi_{2min}}\rvert + \boldsymbol{\Phi_{max}}$	$\lvert\boldsymbol{\Phi_{1min}}\rvert + \boldsymbol{\Phi_{max}}$	0	$\lvert\boldsymbol{\Phi_{2min}}\rvert$
**4**	$\lvert\boldsymbol{\Phi_{2min}}\rvert + \boldsymbol{\Phi_{max}}$	$\boldsymbol{\Phi_{max} - \Phi_{1min}}$	0	$\lvert\boldsymbol{\Phi_{2min}}\rvert$
**5**	NA	NA	0	-0.119

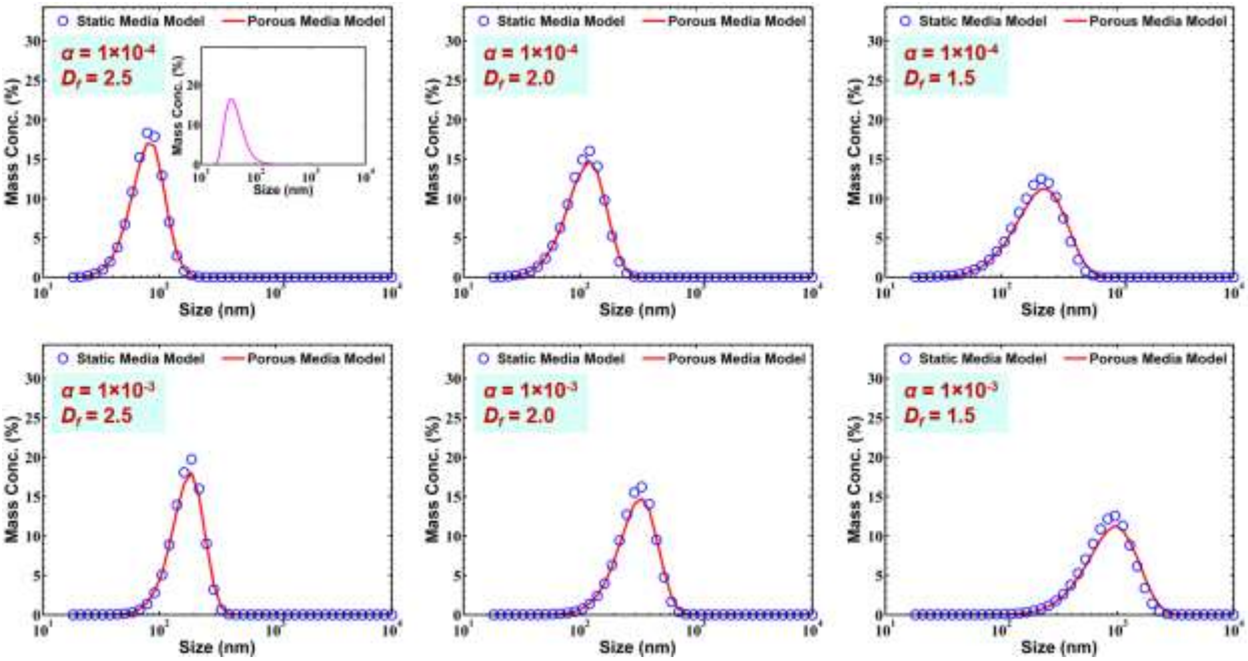

Figure S1. Comparison of the PSDs produced by modified MT3D-USGS model and a previously develop MATLAB code under pure aggregation with different values for attachment efficiency and fractal dimension. The inset illustrates the initial PSD.

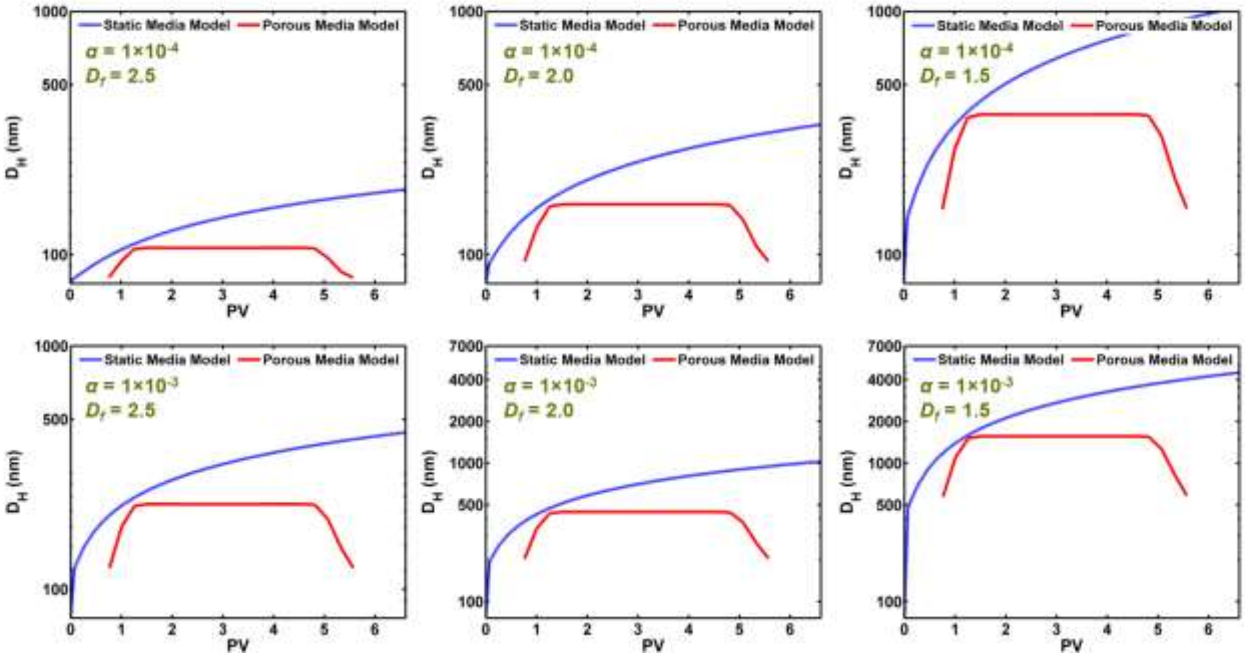

Figure S2. Comparison of the mean hydrodynamic diameter D_H produced by modified MT3D-USGS model and a previously-developed MATLAB code under pure aggregation with different values of attachment efficiency and fractal dimension.

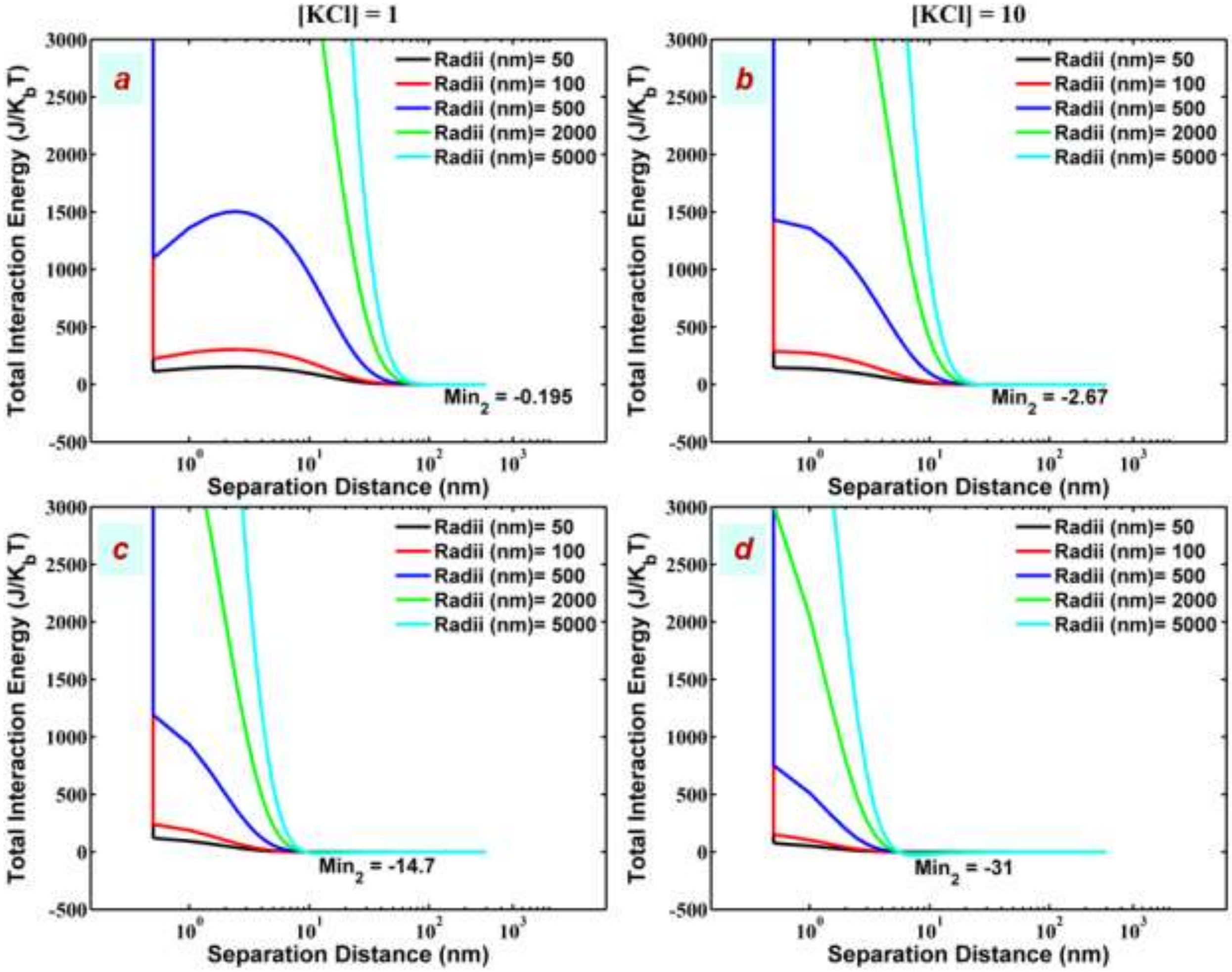

Figure S3. Sphere-plate DLVO interaction energies at various electrolyte concentrations and for different particle sizes at (a) 1 (b) 10 (c) 50, and (d) 100 mM KCl.

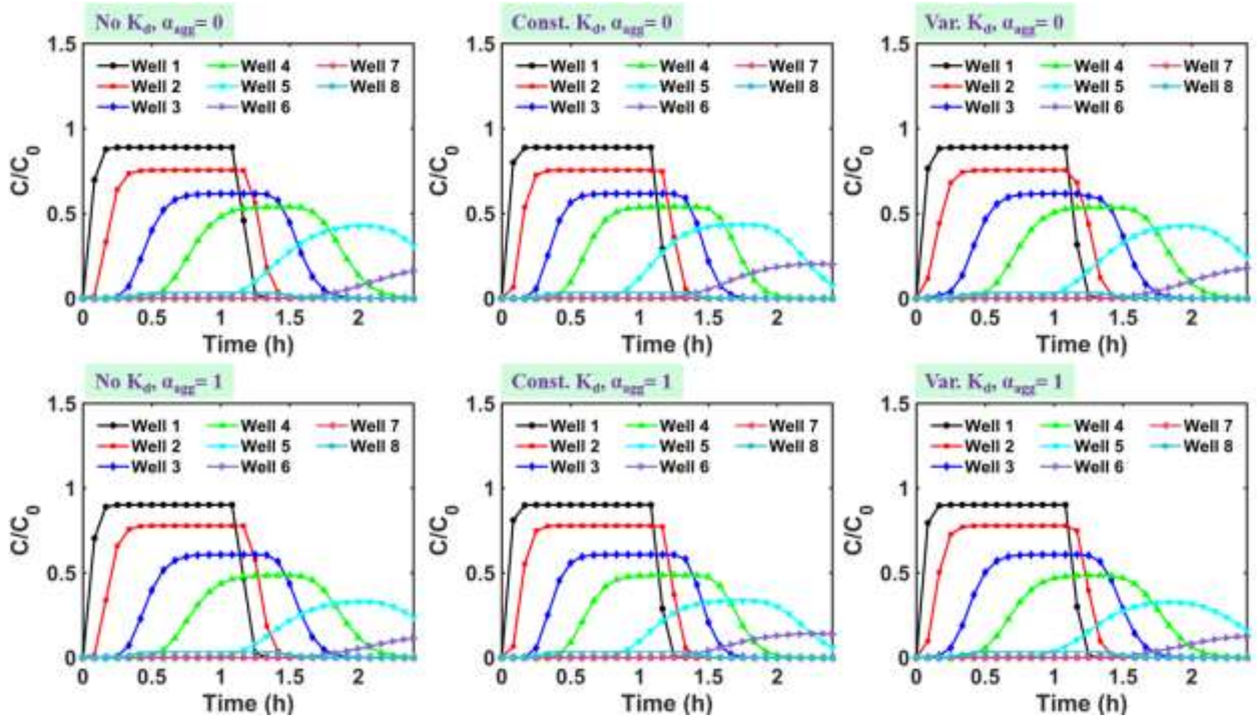

Figure S4. Model-produced BTC at different observation wells in the 3-D simulation domain for various scenarios of considering constant/variable/no acceleration factor and with/without aggregation (α_{agg}= 0 or 1).

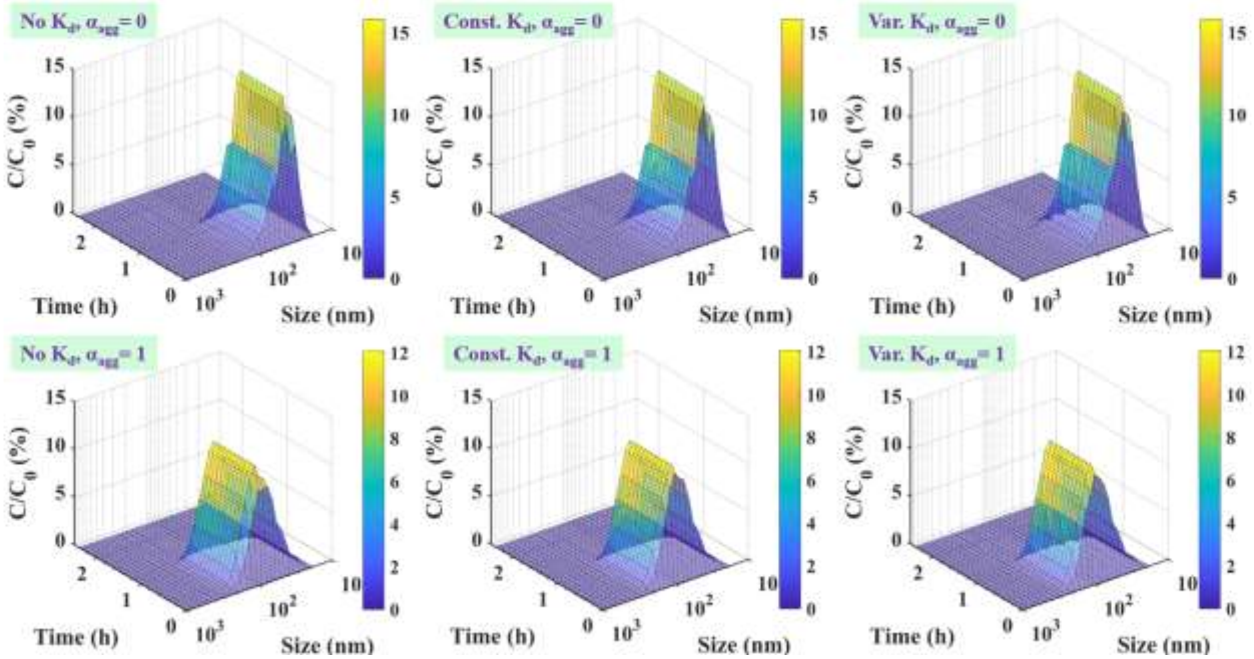

Figure S5. Breakthrough curves vs PSD at Well #1 in the 3-D model domain for different scenarios of aggregation included (α_{agg}=1) or not included (α_{agg}=0) and acceleration not included (no K_d), assumed constant (Const. K_d), or assumed variable (Var. K_d). In this figure size refers to particle diameter.

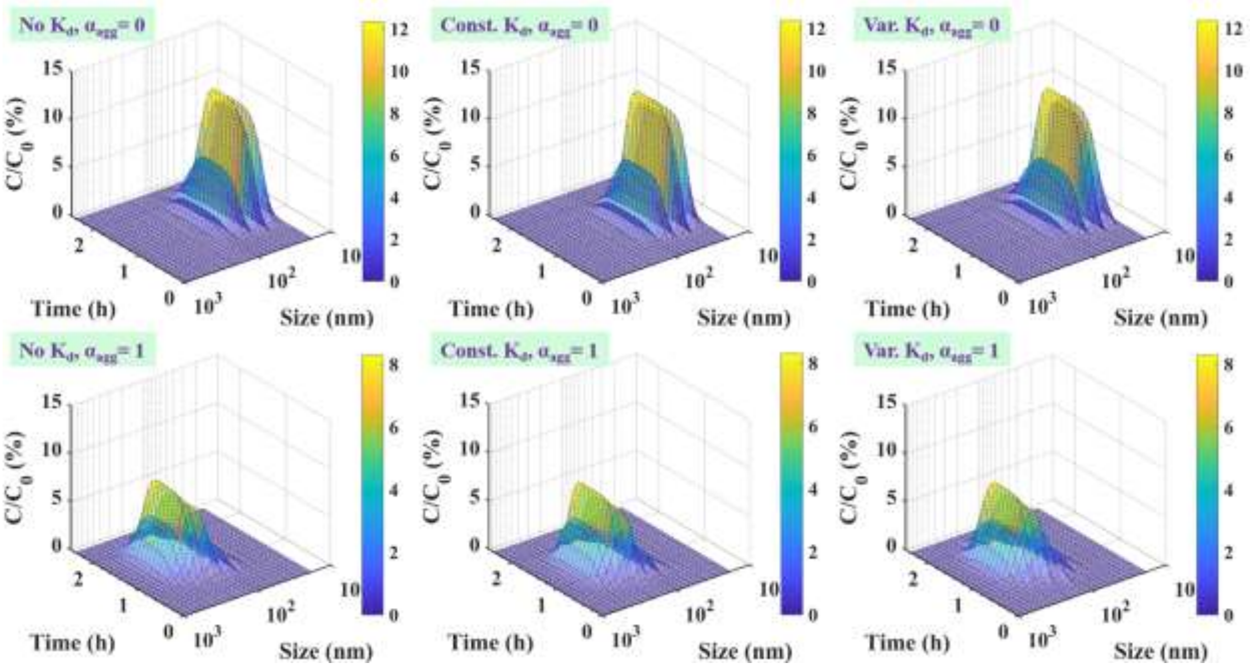

Figure S6. Breakthrough curves vs PSD at well #4 in the 3-D model domain for different scenarios of aggregation included (α_{agg}=1) or not included (α_{agg}=0) and acceleration not included (no K_d), assumed constant (Const. K_d), or assumed variable (Var. K_d). In this figure size refers to particle diameter.

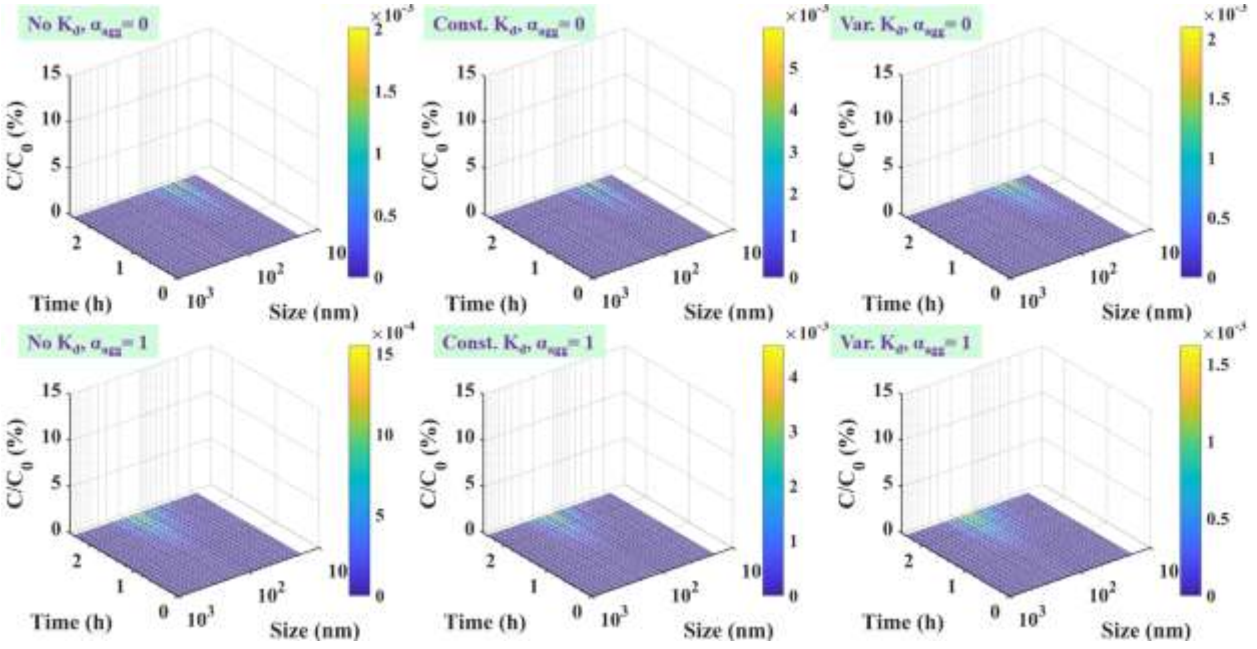

Figure S7. Breakthrough curves vs PSD at Well #7 in the 3-D model domain for different scenarios of aggregation included (α_{agg}=1) or not included (α_{agg}=0) and acceleration not included (no K_d), assumed constant (Const. K_d), or assumed variable (Var. K_d). In this figure size refers to particle diameter.

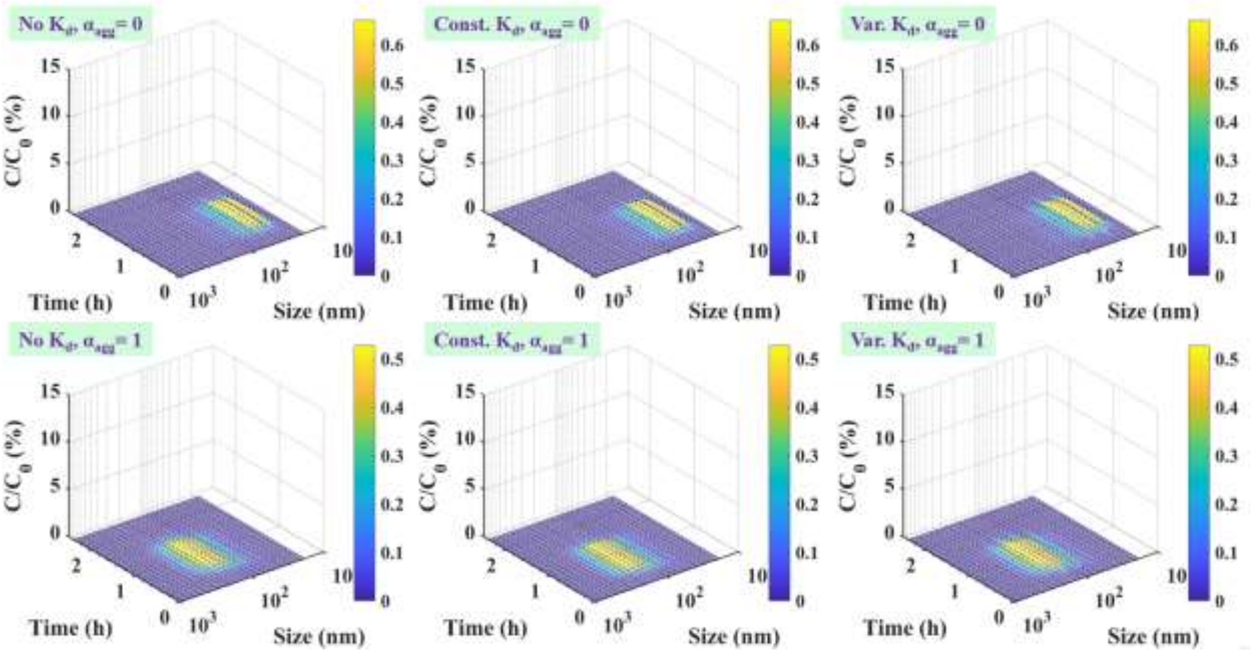

Figure S8. Breakthrough curves vs PSD at Well #8 in the 3-D model domain for different scenarios of aggregation included (α_{agg}=1) or not included (α_{agg}=0) and acceleration not included (no K_d), assumed constant (Const. K_d), or assumed variable (Var. K_d). In this figure size refers to particle diameter.

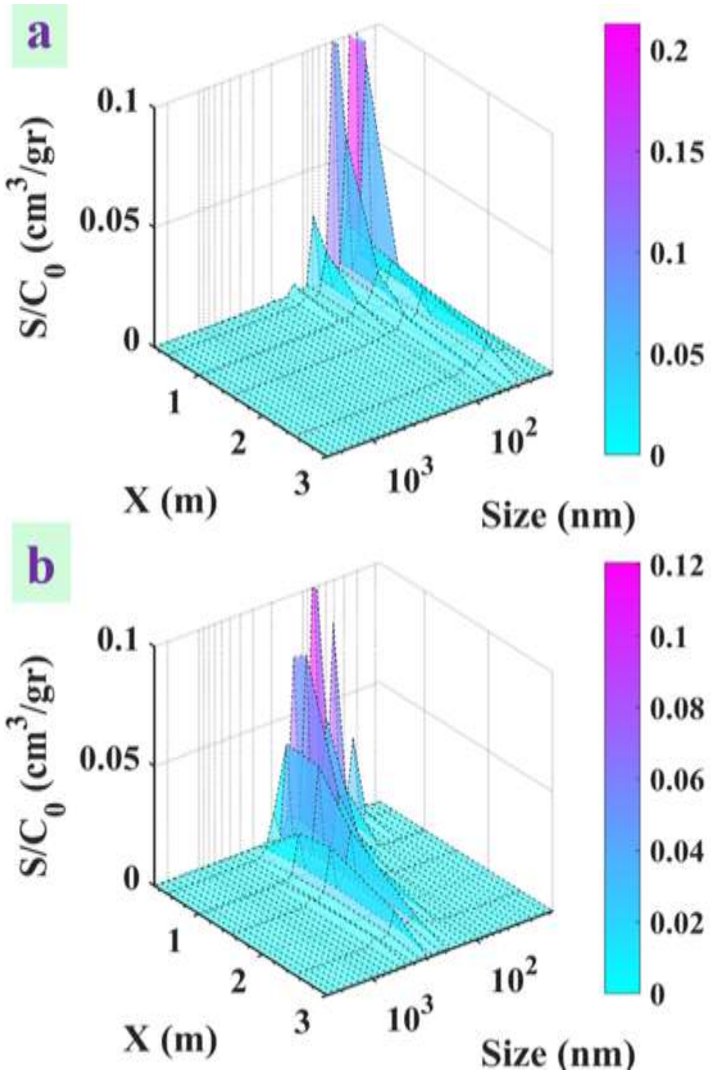

Figure S9. Residual concentration profiles in the direction of flow (at observation wells #1 to #6) without (a) and with (b) incorporating aggregation, α_{agg} = 0 or 1, respectively. Deposition is considered using a size-variable K_{att} calculated using CFT-DLVO. No acceleration factor has been included here.

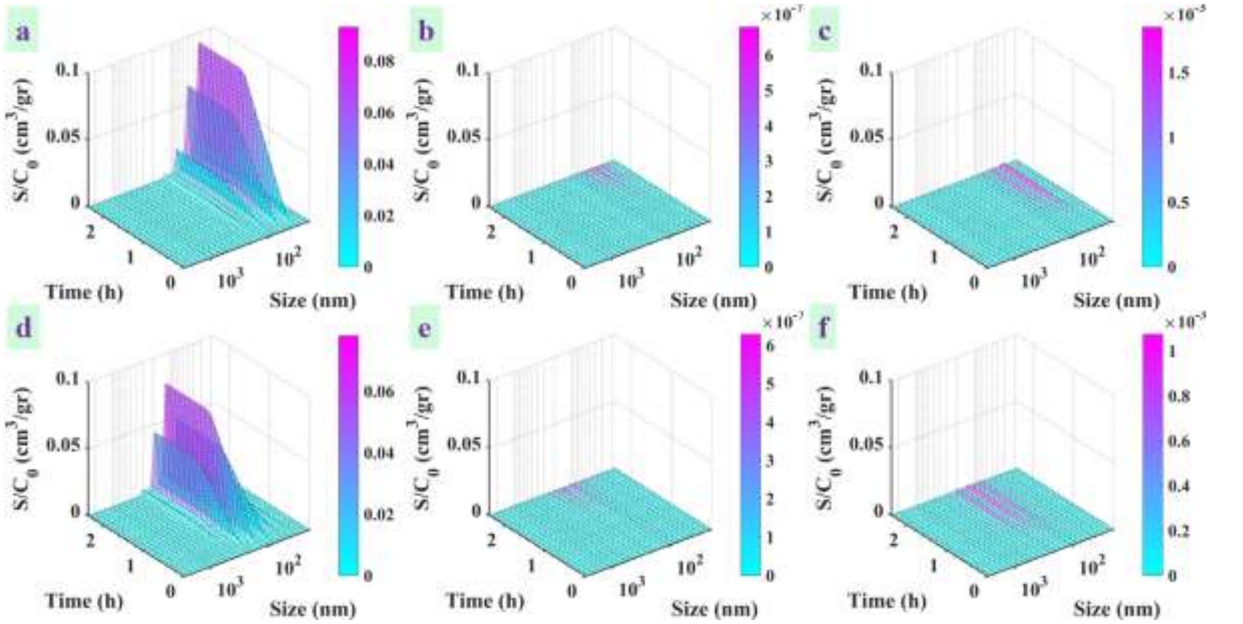

Figure S10. Retained concentration evolution over time versus PSD without (a-c) and with (d-f) incorporating aggregation (α_{agg} = 0 or 1), calculated at well #2 (a,d), well #7 (b,e), and well #8 (c,f). Deposition is considered using a size-variable K_{att} calculated using CFT-DLVO. No acceleration factor has been included here.

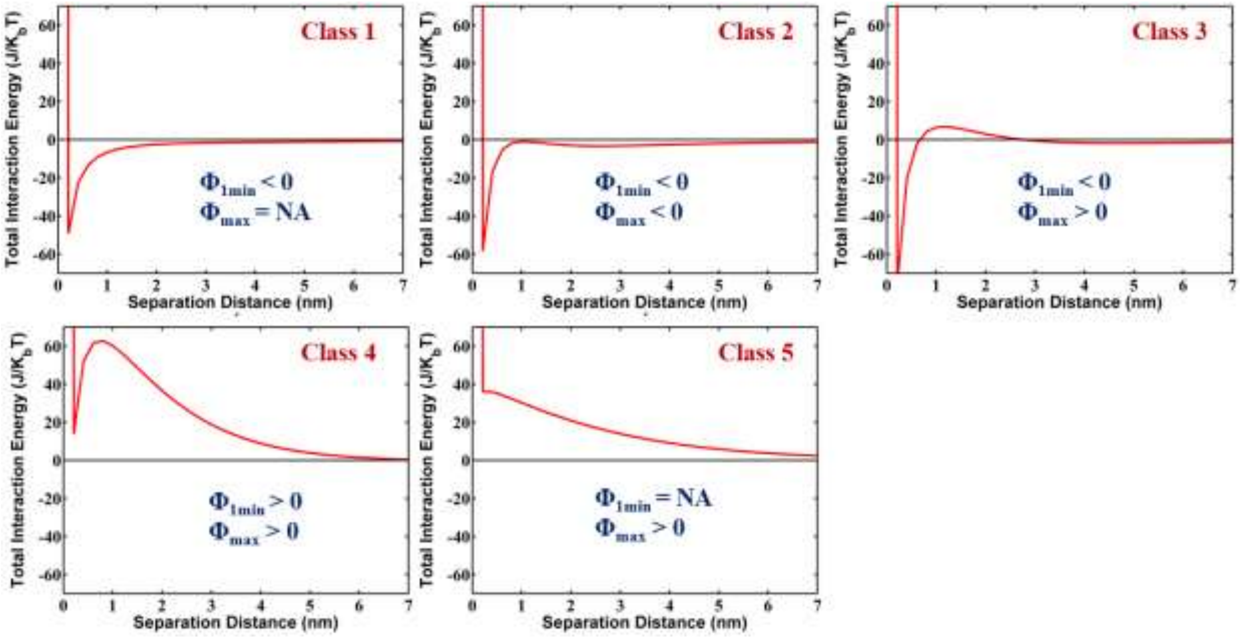

Figure S11. Five categories of possible interaction energy profile that can happen for favourable and unfavourable interaction conditions evaluated using primary minimum, Φ_{1min}, secondary minimum, Φ_{2min}, and the energy barrier, Φ_{max}. The profiles have been produced using a MATLAB code developed for plotting the interaction energy profiles of extended DLVO by arbitrarily varying zeta potentials, particle radius, and ionic strength.

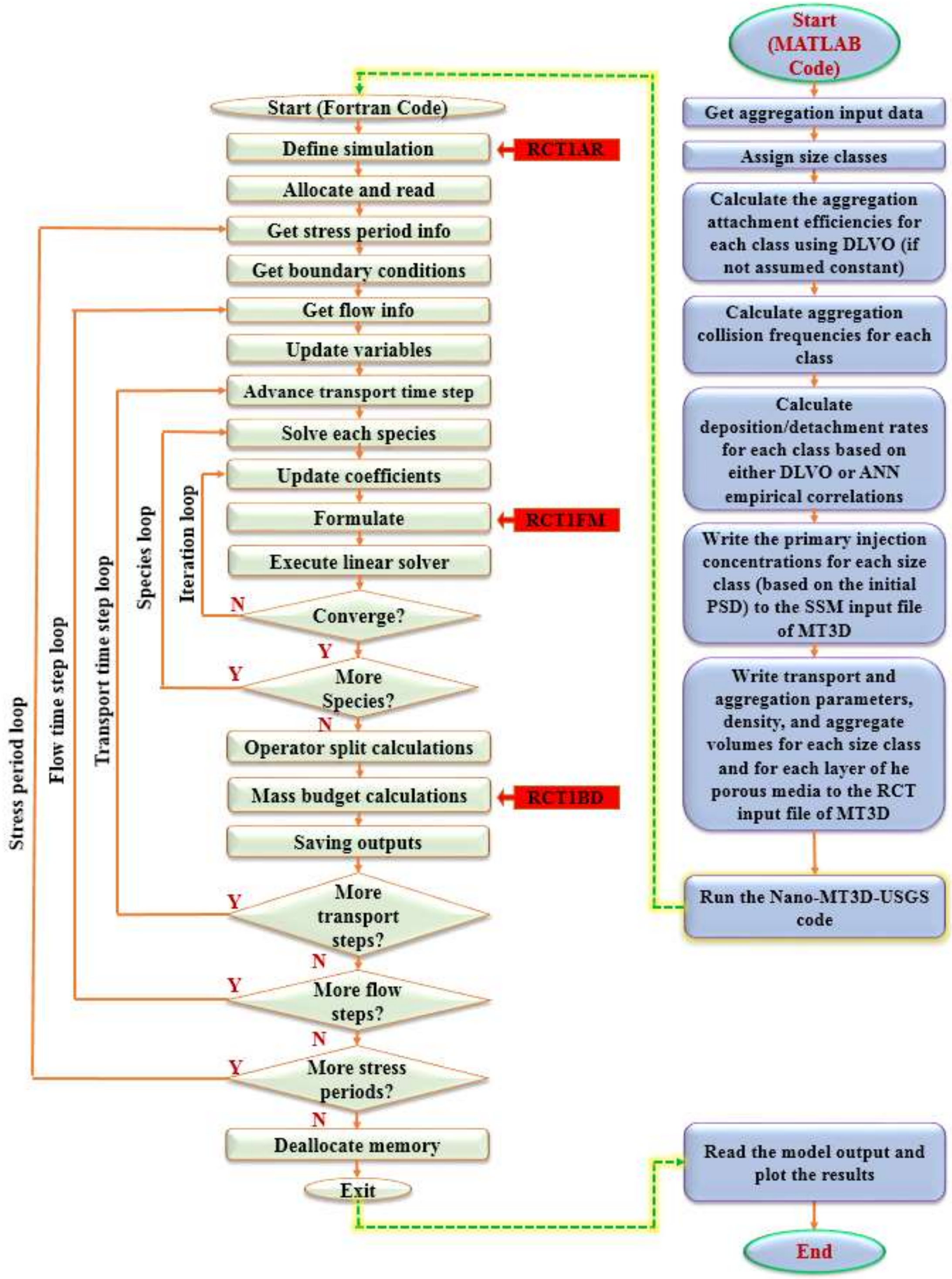

Figure S12. The flowcharts of the integrated FP technique in the USGS-MT3D code which is controlled by a MATLAB code. The subroutine names shown in red boxes are those which are modified in the original MT3D-UGS code. Blue charts are related to the MTALAB code while green charts belong the Fortran code.

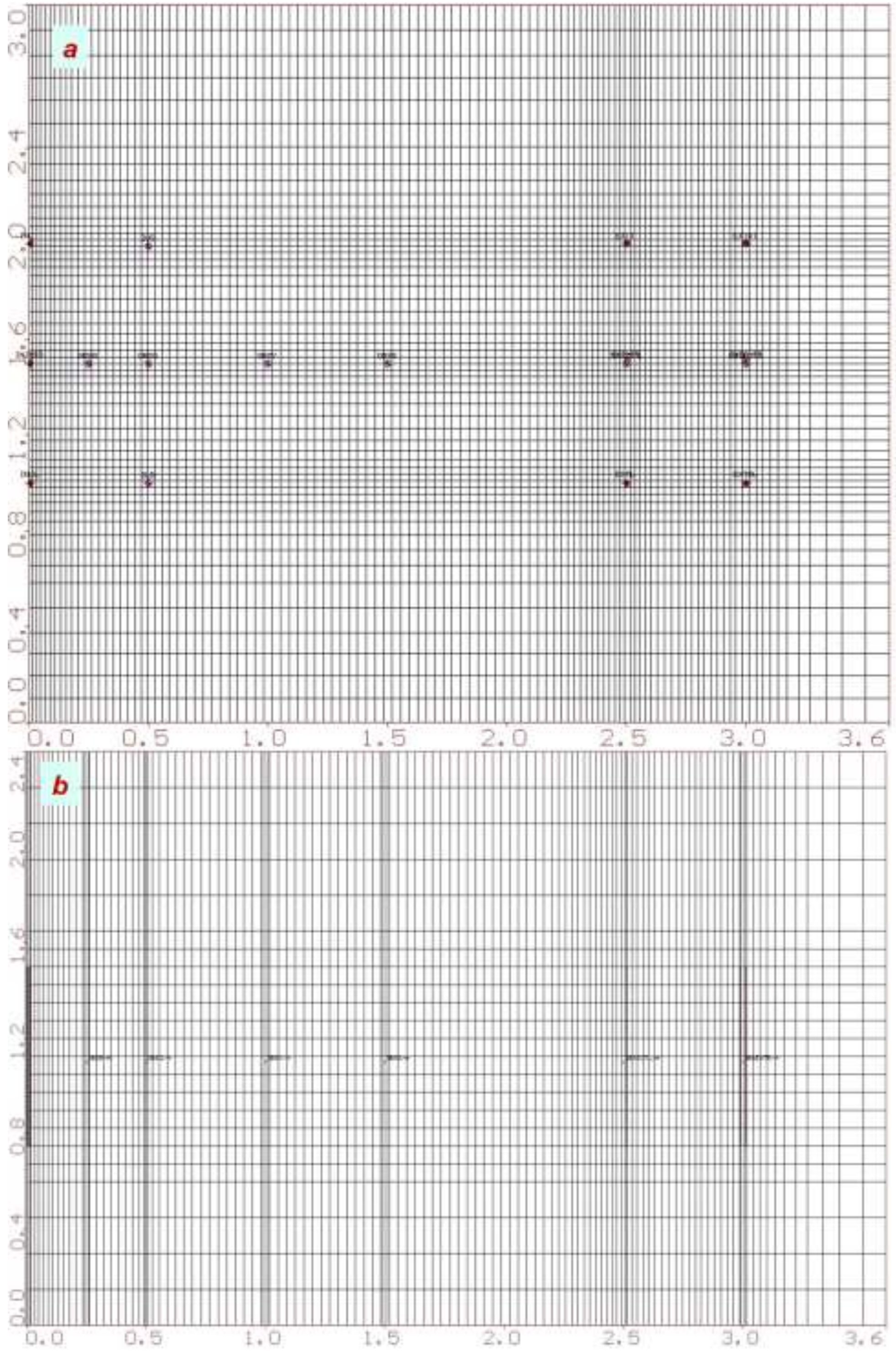

Figure S13. Space discretization of the 3-D model domain simulation for finite difference solution of the coupled aggregation and transport problem. (a) plan view of the domain; (b) side view of the domain in a cross section along the middle row of observation wells.

References

1 Babakhani, P., Bridge, J., Doong, R.-a. & Phenrat, T. Continuum-based models and concepts for the transport of nanoparticles in saturated porous media: A state-of-the-science review. *Adv. Colloid Interface Sci.* **246**, 75-104, doi:https://doi.org/10.1016/j.cis.2017.06.002 (2017).

2 Babakhani, P., Fagerlund, F., Shamsai, A., Lowry, G. V. & Phenrat, T. Modified MODFLOW-based model for simulating the agglomeration and transport of polymer-modified Fe nanoparticles in saturated porous media. *Environ Sci Pollut Res, 1-20, doi:10.1007/s11356-015-5193-0*, doi:10.1007/s11356-015-5193-0 (2018).

3 Zheng, C. & Wang, P. P. A modular three-dimensional multi-species transport model for simulation of advection, dispersion and chemical reactions of contaminants in groundwater systems; documentation and user's guide. *US Army Engineer Research and Development Center Contract Report SERDP-99-1, Vicksburg, Mississippi, USA* (1999).

4 Becker, M. D. *et al.* In situ measurement and simulation of nano-magnetite mobility in porous media subject to transient salinity. *Nanoscale* **7**, 1047-1057 (2015).

5 Bai, C. & Li, Y. Modeling the transport and retention of nC 60 nanoparticles in the subsurface under different release scenarios. *J. Contam. Hydrol.* **136**, 43-55 (2012).

6 Elimelech, M., Gregory, J. & Jia, X. *Particle deposition and aggregation: measurement, modelling and simulation*. (Butterworth-Heinemann, 1998).

7 Dale, A. L., Lowry, G. V. & Casman, E. A. Accurate and fast numerical algorithms for tracking particle size distributions during nanoparticle aggregation and dissolution. *Environmental Science: Nano* **4**, 89-104 (2017).

8 Hunt, J. R. Self-similar particle-size distributions during coagulation: theory and experimental verification. *J. Fluid Mech.* **122**, 169-185 (1982).

9 Veerapaneni, S. & Wiesner, M. R. Hydrodynamics of fractal aggregates with radially varying permeability. *J. Colloid Interface Sci.* **177**, 45-57 (1996).

10 Vikesland, P. J., Rebodos, R. L., Bottero, J. Y., Rose, J. & Masion, A. Aggregation and sedimentation of magnetite nanoparticle clusters. *Environmental Science: Nano* **3**, 567-577 (2016).

11 Thill, A., Moustier, S., Aziz, J., Wiesner, M. R. & Bottero, J. Y. Flocs restructuring during aggregation: experimental evidence and numerical simulation. *J. Colloid Interface Sci.* **243**, 171-182 (2001).

12 Jeldres, R. I., Concha, F. & Toledo, P. G. Population balance modelling of particle flocculation with attention to aggregate restructuring and permeability. *Adv. Colloid Interface Sci.* **224**, 62-71 (2015).

13 Aziz, J. J., Serra, C. A. & Wiesner, M. R. Hydrodynamics of permeable aggregates in differential sedimentation. *Environmental engineering science* **20**, 21-31 (2003).

14 Neale, G., Epstein, N. & Nader, W. Creeping flow relative to permeable spheres. *Chem. Eng. Sci.* **28**, 1865-1874 (1973).

15 Babakhani, P., Doong, R.-a. & Bridge, J. Significance of early and late stages of coupled aggregation and sedimentation in the fate of nanoparticles: measurement and modelling. *Environmental science & technology* **doi: 10.1021/acs.est.7b05236** (2018).

16 Gregory, J. *Particles in water: properties and processes*. (CRC Press, 2005).

17 Lee, D. G., Bonner, J. S., Garton, L. S., Ernest, A. N. S. & Autenrieth, R. L. Modeling coagulation kinetics incorporating fractal theories: a fractal rectilinear approach. *Water Res.* **34**, 1987-2000 (2000).

18 Sterling, M. C., Bonner, J. S., Ernest, A. N. S., Page, C. A. & Autenrieth, R. L. Application of fractal flocculation and vertical transport model to aquatic sol–sediment systems. *Water Res.* **39**, 1818-1830 (2005).

19 Yao, K.-M., Habibian, M. T. & O'Melia, C. R. Water and waste water filtration. Concepts and applications. *Environmental Science & Technology* **5**, 1105-1112, doi:10.1021/es60058a005 (1971).

20 Tufenkji, N. & Elimelech, M. Correlation equation for predicting single-collector efficiency in physicochemical filtration in saturated porous media. *Environmental Science & Technology* **38**, 529-536 (2004).

21 Bradford, S. A. & Torkzaban, S. Determining parameters and mechanisms of colloid retention and release in porous media. *Langmuir* **31**, 12096-12105 (2015).

22 Babakhani, P., Bridge, J., Doong, R.-a. & Phenrat, T. Parameterization and prediction of nanoparticle transport in porous media: A reanalysis using artificial neural network. *Water Resour. Res.* **53**, 4564-4585, doi:10.1002/2016WR020358 (2017).

23 Li, J., Xie, X. & Ghoshal, S. Correlation Equation for Predicting the Single-Collector Contact Efficiency of Colloids in a Horizontal Flow. *Langmuir* **31**, 7210-7219 (2015).

24 Phenrat, T., Kim, H.-J., Fagerlund, F., Illangasekare, T. & Lowry, G. V. Empirical correlations to estimate agglomerate size and deposition during injection of a polyelectrolyte-modified Fe0 nanoparticle at high particle concentration in saturated sand. *J. Contam. Hydrol.* **118**, 152-164, doi:10.1016/j.jconhyd.2010.09.002 (2010).

25 Gregory, J. Approximate expressions for retarded van der Waals interaction. *J. Colloid Interface Sci.* **83**, 138-145 (1981).

26 Wang, D. *et al.* Humic acid facilitates the transport of ARS-labeled hydroxyapatite nanoparticles in iron oxyhydroxide-coated sand. *Environmental science & technology* **46**, 2738-2745 (2012).

27 Hogg, R., Healy, T. W. & Fuerstenau, D. W. Mutual coagulation of colloidal dispersions. *Transactions of the Faraday Society* **62**, 1638-1651 (1966).

28 Zhang, W. in *Nanomaterial Impacts on Cell Biology and Medicine* (ed Yongsheng Chen David G. Capco) Ch. 2, 19-43 (Springer, 2014).

29 Inyang, M. *et al.* Filtration of engineered nanoparticles in carbon-based fixed bed columns. *Chem. Eng. J.* **220**, 221-227 (2013).

30 Tian, Y., Gao, B., Silvera-Batista, C. & Ziegler, K. J. Transport of engineered nanoparticles in saturated porous media. *J. Nanopart. Res.* **12**, 2371-2380 (2010).

31 Li, K. & Chen, Y. Effect of natural organic matter on the aggregation kinetics of CeO 2 nanoparticles in KCl and CaCl 2 solutions: measurements and modeling. *J. Hazard. Mater.* **209**, 264-270 (2012).

32 Wang, D. *et al.* Transport behavior of humic acid-modified nano-hydroxyapatite in saturated packed column: effects of Cu, ionic strength, and ionic composition. *J. Colloid Interface Sci.* **360**, 398-407 (2011).

33 Johnson, R. L. *et al.* Field-Scale Transport and Transformation of Carboxymethylcellulose-Stabilized Nano Zero-Valent Iron. *Environmental science & technology* **47**, 1573-1580 (2013).
